# Single transcription factor efficiently leads human induced pluripotent stem cells to functional microglia

**DOI:** 10.1186/s41232-022-00201-1

**Published:** 2022-07-01

**Authors:** Iki Sonn, Fumiko Honda-Ozaki, Sho Yoshimatsu, Satoru Morimoto, Hirotaka Watanabe, Hideyuki Okano

**Affiliations:** 1 Department of Physiology, Keio University School of Medicine, Tokyo, 160-8582 Japan; 2 Research Fellow of Japan Society for the Promotion of Science (JSPS), Tokyo, 102-0083 Japan; 3 K Pharma, Inc., Fujisawa, Kanagawa 251-8555 Japan; 4 Department of Pediatrics and Developmental Biology, Tokyo Medical and Dental University, Tokyo, 113-8510 Japan

**Keywords:** Human pluripotent stem cells, Microglia, *SPI1*, PU.1

## Abstract

**Background:**

Microglia are innate immune cells that are the only residential macrophages in the central nervous system. They play vital physiological roles in the adult brain and during development. Microglia are particularly in the spotlight because many genetic risk factors recently identified for neurodegenerative diseases are largely expressed in microglia. Rare polymorphisms in these risk alleles lead to abnormal activity of microglia under traumatic or disease conditions.

**Methods:**

In the present study, to investigate the multifaceted functions of human microglia, we established a novel robust protocol to generate microglia from human induced pluripotent stem cells (hiPSCs) using a combination of cytokines and small chemicals essential for microglia ontogeny. Moreover, we highly enhanced the microglial differentiation efficiency by forcing the expression of PU.1, a crucial transcription factor for microglial development, during posterior mesoderm differentiation.

**Results:**

By our novel method, we demonstrated the generation of a greater number of hiPSC-derived microglia (hiMGLs, approximately 120-folds) than the prior methods (at most 40-folds). Over 90% of the hiMGLs expressed microglia-specific markers, such as CX3CR1 and IBA-1. Whole-transcriptome analysis revealed that these hiMGLs are similar to human primary microglia but differ from monocytes/macrophages. Furthermore, the specific physiological functions of microglia were confirmed through indices of lipopolysaccharide responsiveness, phagocytotic ability, and inflammasome formation. By co-culturing these hiMGLs with mouse primary neurons, we demonstrated that hiMGLs can regulate the activity and maturation of neurons.

**Conclusions:**

In this study, our new simple, rapid, and highly efficient method for generating microglia from hiPSCs will prove useful for future investigations on microglia in both physiological and disease conditions, as well as for drug discovery.

**Supplementary Information:**

The online version contains supplementary material available at 10.1186/s41232-022-00201-1.

## Background

Microglia, the unique immune cell type in the brain parenchyma, account for 5–15% of total cells in the central nervous system (CNS) and play critical roles in adult brain homeostasis but also in neural circuit formation during brain development [1]. In the brain, microglia ramified processes reaching up to 100 μm, continuously sense the alteration of their surrounding environment [2, 3]. During development, microglia enter the CNS during neurogenesis. At the prenatal stage, microglia are known to induce neuronal cell death, promote neuronal facilitation, and limit axon outgrowth, while they promote synapse maturation and remodel neural networks after birth [4]. Depletion of microglia during development is reported to disrupt the development of functional neurons, through the reduction of spine size and excitatory synaptic inputs [5]. Furthermore, several studies have demonstrated that besides their action on neurons, microglia also contribute to the differentiation of glial cells (oligodendrocytes and astrocytes) by secreting proinflammatory cytokines [6, 7]. In the adult brain, microglia also have a pivotal role in pathogen defense and clearance of cellular debris. In addition, microglia are also important in regulating the survival and differentiation of oligodendrocyte progenitor cells (OPCs) [8], thereby supporting adult neurogenesis in neurogenic areas and neuronal activity [9]. Finally, microglia are known to eliminate presumably weak synapses during sleep. These findings support the idea that microglia have versatile physiological functions in the adult brain [10].

Microglia has recently drawn an increasing interest because of their recently recognized implication in several neurological diseases. A number of genes specifically expressed in microglia has been identified as causative factors of several brain disorders and neurodegenerative diseases, especially Alzheimer’s disease (AD) and amyotrophic lateral sclerosis (ALS) [11]. Therefore, it is urgent to understand the cellular biology of microglia under physiological and disease conditions. Considering species differences and a limited accessibility to human specimens, there is a thirsty need to explore a new resource of human microglia [12]. As human induced pluripotent stem cells (hiPSCs) have opened the possibility to develop new methods for studying human neural cells in multiple contexts, several protocols for deriving microglia from hiPSCs have been published thus far [13–17]. All of them are basically established on cytokine combinations requiring an extended duration of differentiation, followed by a purification (cell sorting) of the microglial progenitor cells generated. Therefore, more efficient and cost-effective protocols for generating human microglia remain to be unearthed. In contrast to other ectoderm-derived neural cell types, microglia are derived from myeloid progenitor cells in the yolk sac, which appear in the extra-embryonic mesoderm [18–20]. Myeloid cells leave the blood island in the yolk sac and then enter the embryonic brain at the onset of blood circulation and colonize the neuroepithelium before blood-brain barrier formation.

Here, we show that a large number of human microglia-like cells (hiMGLs) can be derived from hiPSCs by a novel method, using overexpression of PU.1, the pivotal transcription factor during microglia development [21]. These hiMGLs showed extremely similar features of primary microglia as scrutinized by transcriptome analysis and exhibited characteristics closer to in vivo microglia when co-cultured with mouse primary neurons. Finally, our novel method provides a great opportunity to study the physiological functions of human microglia in the healthy CNS, as well as in the context of neurodegenerative diseases.

## Methods

All reagents used in this study are summarized in Supplementary Table S1.

### Cell culture

#### Maintenance and culture of human induced pluripotent stem cells (hiPSCs)

The human iPSC lines 201B7 [22], WD39 [23], and RPC802 (ReproCell Inc., cat# RCRP002N) were cultured as feeder-free cultures. All iPSCs were maintained in 12-well plates (Corning) coated with iMatrix-511 in feeder-free condition. hiPSCs were cultured in StemFit/AK02N (Ajinomoto) medium supplemented with penicillin/streptomycin in a humidified incubator (37 °C, 5% CO_2_). hiPSCs were routinely passaged every week, and the medium was changed every 2 days.

All procedures are following the instruction of the protocol (Human iPS cell culture under feeder-free conditions) published by CiRA (https://www.cira.kyoto-u.ac.jp/j/research/img/protocol/ )[22].

#### Establishment of hiPSC clones with inducible PU.1 overexpression

Genejuice (Sigma-Aldrich, 70967) was used as a transfection reagent. Nine microliters of Genejuice mixed with 200 μl Opti-MEM medium (Gibco™, 31985070) was incubated at room temperature for 5 min; the plasmids were then added (pCMV-HyPBase_PGK-Puro 0.4 μg, pG-PB-CAG-rtTA3G-IH 0.4 μg, PB-tet-PHS-*SPI1* 0.8 μg) followed by another 5-min incubation at room temperature.

Feeder-free hiPSCs were treated with Y-27632 (Wako, 253-00511) (10 μM in AK02N) 1 h before the following procedure: hiPSCs were detached with TrypLE Select (Life Technologies, A12859-01) (0.5×, incubate for 10 min at 37 °C); 50,000 cells were counted using Trypan Blue (after being spun down at 1000 rpm for 5 min).

After centrifugation, the supernatant was removed carefully and GeneJuice-DNA mix was added, incubated for 10 min at room temperature. Cells were then seeded evenly into 6 wells of a 12-well plate. The plate was poured with 0.8 ml AK02N medium supplemented with penicillin/streptomycin, 10 μM Y-27632, and iMatrix-511. The medium was changed on the next day to remove Y-27632, while hygromycin (20 μg/ml) was supplemented for the selection of hiPSCs. When the colonies had grown larger, puromycin (2 μg/ml) was added to the medium. The final concentration of hygromycin was 200 μg/ml, while that of puromycin was 10 μg/ml. Colonies were then mechanically isolated using P10 tips under a microscope. Isolated colonies were seeded into a 24-well plate individually, with a medium supplemented with Y-27632 and iMatrix-511.

#### Human iPSC-derived microglia (hiMGL) differentiation

The basic protocol of hiMGL generation (CK protocol) was modified based on several published articles (36, 37). Briefly, the generation of microglia cells from iPSCs is divided into two stages. The first stage is the generation of hematopoietic progenitor cells from hiPSCs (hiHPCs; ∼ day 18), and the second is the differentiation of microglia-like cells from hiHPCs (day 19∼).

Day 0: hiPSCs in 6-well plates were washed with PBS once. Then, 2 ml hiHPC diff. medium supplied with BMP4 (PEPROTECH, 20 ng/ml) and CHIR99021 (Focus Biomolecules, 2 μM) was added to each well. Cells were placed in a hypoxia incubator (5% O_2_, 5% CO_2_, 37 °C) until day 6. We noted that the size of colonies seems to affect the efficiency of differentiation, with colonies smaller than usually used for passage resulting in optimal hiMGL differentiation.

Day 2: The whole medium was replaced by 2 ml fresh medium supplied with BMP4 (20 ng/ml), VEGF (Thermo Fisher Scientific, 50 ng/ml), and FGF2 (PEPROTECH, 20 ng/ml).

Day 4: The whole medium was replaced by 2 ml fresh medium supplied with VEGF (15 ng/ml) and FGF2 (5 ng/ml).

Day 6: Cells were collected into 15-ml tubes and centrifuged at 1000 rpm for 5 min. Half of the medium was removed, while the remaining medium (about 1 ml) and cells were returned back to the well. One milliliter of fresh hiHPC medium supplied with VEGF (30 ng/ml), FGF2 (10 ng/ml), SCF (PEPROTECH, 100 ng/ml), IL-3 (PEPROTECH, 60 ng/ml), IL-6 (PEPROTECH, 20 ng/ml), and IWR-1e (Thermo Fisher Scientific, 5 μM) was added into each well. From this point, cells were placed in a normoxia incubator (20% O_2_, 5% CO_2_, 37 °C).

Day 9: Cells were supplemented with 1 ml medium as described in day 6.

Day 12: Full volume of medium containing floating cells was collected into 15-ml tubes and centrifuged at 1000 rpm for 5 min. The half volume of the supernatant was carefully removed. Another half of the medium containing all the cells was returned back to the well. Two milliliters of hiHPC medium supplemented with FGF2 (10 ng/ml), SCF (100 ng/ml), IL-3 (60 ng/ml), and IL-6 (20 ng/ml) was added into each well.

Day 15: Cells were supplemented with 1 ml medium as described in day 12.

Day 18: Cells were collected into Falcon tubes and centrifuged at 300×*g* for 5 min. Then, the supernatant was removed carefully and thoroughly. Cells were seeded at 40,000–50,000 cells in 0.8 ml hiMGL diff. medium supplemented with five cytokine cocktail (IL-34 100 ng/ml, TGFβ1 50 ng/ml, M-CSF 25 ng/ml, CD200 100 ng/ml, and CX3CL1 100 ng/ml, all from PEPROTECH) each well in 12-well plates. Plates were coated with poly-d lysine (PDL, R&D system) for at least 1 h in advance and washed with PBS three times before use.

Day 19: Half volume of hiMGL diff. medium supplemented with five cytokine cocktails was added into hiMGL culture. After day 19, the half volume of the medium was changed every 2 days. hiMGL was cultured for another week could be used for analysis.

hiHPC diff. medium: StemPro™-34 SFM (1X) (Gibco™, 10639011), GlutaMax (Gibco™, 1X), holo-transferrin (Nacalai tesque, 200 μg/ml), l-ascorbic acid (Sigma-Aldrich, 500 μM), monothioglycerol (Sigma-Aldrich, 450 μM), and penicillin/streptomycin, filtered through a 0.45-μm filter.

hiMGL diff. medium: DMEM/F12 (1:1, Gibco™), GlutaMax (1×), NEAA (Thermo Fisher Scientific, 1×), B27 (Thermo Fisher Scientific, 1×), N2 (Thermo Fisher Scientific, 0.5×), ITS-G (Thermo Fisher Scientific, 1×), additional insulin (Sigma-Aldrich, 5 μg/ml), monothioglycerol (200 μM), and penicillin/streptomycin, filtered through a 0.45-μm filter. DMEM can be replaced by the same volume of IMDM (Gibco™).

#### hiPSC-derived microglia (hiMGL) generation by overexpression of *PU.1*

The same procedure as usual, except that doxycycline (1 μg/ml) was added into the medium during days 6∼18.

#### Mouse primary hippocampal and cerebellar neurons culture

Mouse hippocampus and cerebellum were dissected from P0-P2 wild-type C57BL/6JJcl mouse (CLEA Japan, Inc.) brain as previously described [24, 25]. In brief, the dissected brain tissue was dissociated with Trypsin-EDTA (Sigma-Aldrich) for 12 min and triturated with a fire-polished glass pipette. Neural cells were then plated at a density of 5,000,000 cells/ml on 10-mm cover glasses in 48-well plate with 400 μl plating medium (80% DMEM, 10% F12, 10% FBS and penicillin/streptomycin). Cover glasses were coated with poly-d-lysine in advance. On the next day, the medium was changed to a neurobasal medium (Thermo Fisher) supplemented with 1% B27 and l-glutamine; half medium was changed every 2–3 days thereafter.

#### Transfection of the plasmid DNA expressing GFP-β-actin and GCaMP6s into mouse primary neurons

Lipofectamine 3000 was used as a transfection reagent, and all procedures were following the manufacturer’s guidelines.

#### Co-culture of hiMGL and mouse primary neurons

hiMGL conditioned medium was collected and centrifuged at 300×*g* for 5 min. The supernatant was carefully transferred into a new tube. hiMGLs were washed with PBS once and incubated at 37 °C for 5 min with 100 μl Accutase. Then, hiMGL were detached and collected using their conditioned medium then centrifuged at 300×*g* for 5 min. hiMGLs were then resuspended in a neurobasal medium supplemented with 2% B27 and poured into a 48-well plate of mouse primary neuron cultures. For immunocytochemistry, cells were co-cultured for 3 days, while for morphological analysis and calcium imaging, cells were co-cultured for a week.

### Flow cytometry analysis

hiMGLs were collected using Accutase and then washed twice by FACS solution (PBS + 1% FBS). Next, cells were stained with primary antibodies for 30 min on ice. For the detection of intracellular antigens such as PU.1, cells were incubated with 0.1% Triton-X100 for 10 min on ice before incubating the primary antibody. After FACS solution wash once, cells were stained 15 min on ice with the secondary antibody, followed by another wash with FACS solution wash. FACS analyses were performed using FACS Verse (BD Biosciences, USA). Antibodies used are summarized in Supplementary Table S2.

### Quantitative reverse transcription polymerase chain reaction (qRT-PCR)

Cells were collected in RLT buffer and then homogenized by QIAshredder (Qiagen #79656). Total RNAs were isolated with RNeasy Mini Kit (Qiagen #74106) following the manufacturer’s guidelines and then reverse-transcribed to cDNA by PrimeScript™ II 1st strand cDNA Synthesis Kit (Takara, #6210A). qPCR was performed using a ViiA 7 Real-Time PCR System (Thermo Fisher Scientific) and using TB Green Premix Ex Tag II (Takara, #RR820). Primers are described in Supplementary Table S3.

### Phagocytosis assay

For phagocytosis assay, microglia cultured for 7 days after replating were transferred onto 10-mm cover glasses in a 48-well plate. Cover glasses were coated with PDL for at least 1 h before use. Cells were placed in the incubator overnight before applying the phagocytosis assay.

Before the phagocytosis assay, the Latex beads and fibrillary amyloid beta were mixed with FBS for 30 min in a 37 °C incubator. Both reagents were diluted one-fifth. Both final concentrations of Latex and fibrillary amyloid beta were 1:1000. Then, 5 μl per vial was added to each well directly. The culture plate was incubated at 37 °C for 1 h, then fixed with 4% PFA following wash twice with PBS and kept in 4 °C for future analysis.

### Immunocytochemistry

Cells were fixed with 4% PFA for 15 min, followed by two PBS washes. For immunocytochemistry, the blocking was done using PBS containing 0.4% Triton X-100 and 1.5% FBS. Primary and secondary antibodies were also diluted in this solution. Cells were incubated with primary antibodies at 4 °C overnight and then washed twice with PBS for 15 min. Incubation with the secondary antibodies lasted 1 h at room temperature. Cells were finally washed twice with PBS and mounted with PermaFluor mounting medium (Thermo Fisher Scientific).

### Western blot

hiMGLs and hiPSCs were lysed in cold RIPA buffer. Complete® Protease Inhibitor and Phosphatase Inhibitor were added freshly to RIPA buffer directly before use. Cell lysates were homogenized by passing through a 1-ml syringe, followed by centrifugation for 30 min at 15,000 rpm at 4 °C. Supernatants were collected and stored at − 80 °C. Protein concentration was determined using a BCA Protein Assay Kit. Ten micrograms of total protein was loaded and separated by 10–20% Extra PAGE gels (Nacalaitesque, Japan) in SDS running buffer. Gels were transferred to nitrocellulose membranes using a Trans-Blot Turbo system. The membranes were blocked with 5% milk in TBST for an hour at room temperature, followed by incubation with α-tubulin antibody or DAP12 antibody at 4 °C overnight. The next day, the membranes were incubated with the corresponding Odyssey secondary antibody diluted in TBST and CanGetSignal buffer (TOYOBO, Japan) (1:1) for an hour at room temperature. Images were captured and analyzed using the Odyssey Imaging system (LI-COR Biosciences, USA).

### Analysis of dendritic spines

After co-culturing with hiMGLs for 2 weeks, primary neurons transfected with GFP-β-actin [26] were immunostained with anti-GFP antibody and captured using a confocal microscope system (Zeiss, LSM700) equipped with a × 63 objective lens (Zeiss, Plan-Apochromat, NA 0.8). Images were analyzed by ImageJ (Ver.2.0.0-rc-69/1.52s).

### Calcium imaging

Calcium imaging was performed as described previously [27]. Primary neurons were transfected with a plasmid expressing GCaMP6s (Addgene plasmid # 51084), and after co-culture with hiMGLs for 2 weeks, calcium images were taken using a IX80 microscopic system (Olympus, Japan) with a 0.75-s step for 4 min. Images were analyzed using ImageJ. Videos were taken by stacks and then merged by Z-projections, and Δ*F*/*F* values were calculated with the fluorescence intensity in the region of interest (usually set on the neuronal cell body).

### Statistics

Statistical analyses and graph creation were done using the GraphPad Prism10 software (Ver.8.2.0). The Mann-Whitney test was used for the statistical analysis of qRT-PCR, western blot, and calcium imaging; the nested *t*-test was used for phagocytosis assay; 2-way ANOVA was used for the analysis of dendrites (*n* = 3 per experiment, 3 independent experiments) (*p < 0.05; **p < 0.01; ***p < 0.001; n.s.: not significant).

### Bulk mRNA-seq analysis

Poly(A)+ RNA was selected and converted to a library of cDNA fragments (mean length, 350 bp) with adaptors attached to both ends for sequencing using the KAPA mRNA Capture Kit (KK8440; Kapa Biosystems), KAPA RNA HyperPrep Kit (KK8542; Kapa Biosystems), KAPA Pure Beads (KK8543; Kapa Biosystems), and SeqCap Adapter Kit A (Roche) according to the manufacturer’s instructions. The cDNA libraries were quantified using the KAPA Library Quantification Kits (KK4828; Kapa Biosystems) and were sequenced using an Illumina HiSeqX to obtain 150-nucleotide sequences (paired-end). Data of mRNA-seq (*fastq* file format) were quality-checked, and low-quality reads (score < 30), adapter sequences, and overrepresented sequences such as poly-A chain were trimmed using the *Trim Galore!* (ver.0.4.0). The remaining reads were mapped to the *Homo sapiens* (hg19) genome using the *Hisat2* (ver.2.2.2.0) [28], and the output file (BAM file format) was summarized using the *featureCounts* (1.5.2) [29]. The summarized data were processed by the *DESeq2* (3.3.0) for estimating their size factors, followed by the removal of reads not expressed in any of the samples. Subsequently, the data were normalized by varianceStabilizingTransformation (vst).

For mRNA-seq analysis, we also included deposited data of previous studies in GEO (https://www.ncbi.nlm.nih.gov/geo/) and DDBJ (https://www.ddbj.nig.ac.jp/) as follows: Monocytes, AH1 iMGL, and adult and fetal microglia in GSE89189 [16].

RNA sequencing data have been deposited in the Gene Expression Omnibus of NCBI (https://www.ncbi.nlm.nih.gov/geo/) under accession no. GSE178284.

## Results

### Determination of crucial cytokines and small molecules for primitive hematopoietic progenitor differentiation

To obtain human microglia-like cells from hiPSCs in vitro, we aimed to develop an efficient protocol using multiple cytokines, according to previously published articles [14–17]. Prior approaches were conceived by the idea that microglia can be differentiated from primitive hematopoietic progenitor cells (HPC), which are derived from the yolk sac originating in the posterior mesoderm [18]. For the induction of primitive HPCs, multiple cytokines, in addition to manipulating the BMP4 and Wnt signaling, were applied in these methods.

To define which signaling pathways are necessary and sufficient for inducing posterior mesoderm formation in vitro, we picked up several candidate reagents (BMP4, CHIR99021, VEGF, TGFβ1, Activin A, SB431542, FGF2) from previous literatures on mesoderm development [30, 31]. Firstly, through immunostaining of mesodermal markers such as brachyury, we confirmed that only CHIR99021 (CHIR), a GSK-3 inhibitor; as well as BMP4, was necessary and sufficient to induce mesoderm-like characters in hiPSCs in vitro (Supplementary Fig. S1a-d). Since VEGF, a key regulator of blood vessel formation and hematopoiesis, and Activin A, which is an agonist of TGF-β/Smad signaling, were applied in multiple protocols for inducing posterior mesoderm from hiPSCs in previous reports [15–17, 32], we next tested if they were necessary for the first 2 days through qRT-PCR. Although the expression of *MSGN1*, a paraxial mesoderm marker, was reduced when Activin A was added, there was no difference in other mesoderm markers [33]. Moreover, the addition of VEGF did not change mesodermal marker genes (Supplementary Fig. S1d). As a result, we decided to limit the additives to BMP4 and CHIR for the first 2 days during the mesodermal differentiation.

Then, during the differentiation to primitive HPCs, we found that besides hematopoietic cells, cardio-lineage cells were also generated simultaneously, which resulted in a lower efficiency of HPC production than expected. According to previous studies, Wnt signaling could be a vital determinant of the balance between the cardiac and hematopoietic lineages [34–36]. Notably, the non-canonical Wnt pathway is beneficial for the myeloid lineage, whereas the canonical Wnt signaling regulates non-myeloid hematopoiesis, including T and B cell development [35, 37, 38]. We first tried two small molecules, IWR-1e and IWP-2, which are both inhibitors of Wnt signaling with distinct mechanisms. IWR-1e is a stabilizer of β-catenin degradation complex, which leads to the specific inhibition of the canonical Wnt pathway, while IWP-2 binds to porcupine to inhibit Wnt secretion and processing and therefore inhibits both canonical and non-canonical Wnt signaling. As measured by qRT-PCR, we found that treatment with IWR-1e during days 6–12 could efficiently induce primitive HPC differentiation (Supplementary Fig. S2). The mRNA expression level of *GYPA*, a specific marker for the primitive hematopoietic lineage (51), which would further differentiate to microglia cells, and *GATA2*, a key transcription factor for the commitment toward the hematopoietic lineage instead of the cardiac lineage [37], was much more upregulated by IWR-1e than IWP-2. Therefore, IWR-1e was included for an efficient primitive hematopoietic lineage induction from day 6. The combination of these cytokines and small molecules (referred to as “CK protocol”) induced the successful differentiation of hiPSCs to primitive HPCs.

### PU.1 overexpression in iPSC-derived mesoderm potently induces microglia progenitor cells

**Fig. 1:**
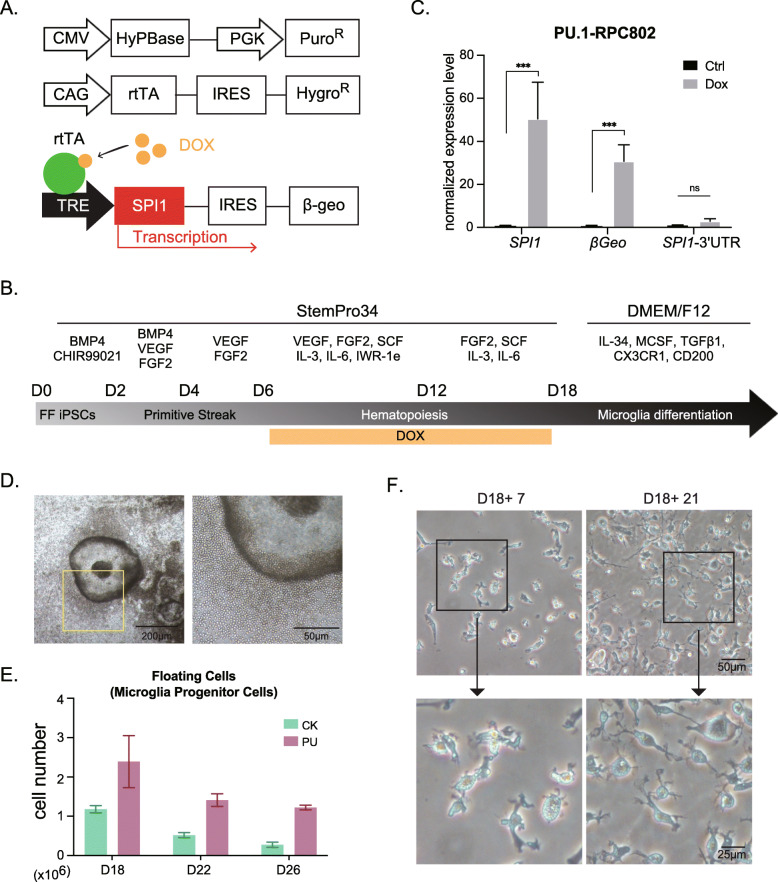
Differentiation of hiMGLs from hiPSCs through overexpression of PU.1 transcription factor. **A** Schematic diagram of the tet-on inducible expression vector of *SPI1* and plasmids expressing hyperactive piggyBac transposase (HyPBase) or reverse tetracycline transactivator (rtTA). **B** Protocol for generating hiMGLs from hiPSCs. For PU protocol, doxycycline (DOX) was added into the medium from days 6 to 18. **C** qRT-PCR data about the expression level 1 day after the DOX was added (day 7). Compared with the groups with no DOX (Ctrl), *SPI1*, as well as congruent *βGeo*, was strikingly upregulated after DOX treatment for 24 h (*n* = 3 independent clones). There was no observed difference of the 3′UTR of the *SPI1* gene, which is not included in the *SPI1* expression plasmid, indicating that the high expression level of *SPI1* on day 7 was exogenous. ns, not significant; * *p* < 0.05; ** *p* < 0.01; *** *p* < 0.001. All data are expressed as mean ± SEM. SEM, standard error of the mean. **D** Bubble-like structures were observed in the PU protocol, not in the CK protocol. Scale bar, 200 μm (left) and 50 μm (right). **E** More than three times of hiHPC (microglia progenitor cells) could be harvested (days 18, 22, 26) by using the PU protocol, compared to the CK protocol (*n* = 3 independent experiments). **F** Representative images of hiMGLs. iMGLs acquired more ramified morphology 3 weeks after re-plating of hiHPCs (day 39) than those from the shorter culture (day 25). Scale bar, 50 μm (upper) and 25 μm (lower)

Although we succeeded in deriving microglia cells from multiple hiPSC lines through the CK protocol, we found an important variability of differentiation efficiency among different hiPSC lines, even between control hiPSCs from healthy individuals. To solve this problem, we next developed a novel method to derive microglia cells from hiPSCs through overexpression of *SPI1*, which encodes PU.1, one of the key transcription factors for microglia development, during hematopoiesis. To enforce the inducible overexpression of PU.1 in hiPSC-derived lineages, we transduced an inducible *SPI1* expression plasmid, as well as plasmids expressing reverse tetracycline transactivator (rtTA) and hyperactive piggyback transposase (Fig. 1A). Following selection with puromycin and hygromycin, we successfully obtained a couple of clones for each of the three independent hiPSC lines used (RPC802, 201B7, and WD39), where all expression plasmids were integrated by a non-viral-based approach.

Because the expression of endogenous *SPI1* mRNA, as well as that of *IRF8* (Supplementary Fig. S3a), another critical transcription factor for microglia differentiation, started at day 6 in CK protocol, we decided to induce PU.1 expression on day 6 in our newly developed protocol (hereafter referred to as “PU protocol”) through addition of doxycycline (Dox) (Fig. 1B). qRT-PCR showed that *SPI1*, as well as *β-geo*, which is a congruent transcript of *SPI1* (Fig. 1A), was increased more than 50 times in Dox-treated cells on day 7, compared with those without Dox treatment (Fig. 1C). In contrast, the mRNA level of the 3′UTR of *SPI1*, which was not included in the PU.1 expression plasmid, was barely changed following Dox treatment, confirming that the increased expression of *SPI1* was derived from the exogenous, not endogenous, *SPI1* transcript (Fig. 1C). A similar induction pattern of *SPI1* and *β-geo* mRNAs was observed in two other hiPSC lines, 201B7 and WD39, upon Dox treatment (Supplementary Fig. S3b). Since the subsequent differentiation was comparable among these iPSC lines carrying the inducible *SPI1* gene, we mostly used RPC802 hiPSCs in the following analyses.

About 1 week after Dox treatment, extruding bubble-like structures, from which a large amount of HPCs (also putative microglia progenitor cells, hereafter hiHPCs), could be observed more frequently in PU protocol (Fig. 1D, Supplementary Fig. S4). Interestingly, this kind of multicellular structure was barely observed in the CK protocol (Fig. 1D, Supplementary Fig. S4). The number of hiHPCs in the culture medium upon PU protocol was more than double compared with that in the CK protocol. Furthermore, those floating cells could be collected during more than three consecutive days in PU protocol, whereas such cells could be collected only once or twice in the CK protocol (Fig. 1E). Moreover, an extended culture with CK protocol led to an extremely small cell number, associated with a morphological change of microglia progenitors as well. These results revealed a clear advantage of PU protocol, compared with the CK protocol.

After re-plated to culture dishes for another week (day 25), the hiHPCs started to show microglia-like morphology, and further long culture led to a more ramified shape at day 39, similar to homeostatic sensing microglia (Fig. 1F). Hereafter, the terminal differentiated microglia-like cells in the PU protocol were referred to as (PU-) human induced microglia-like cells (hiMGLs).

### High purity PU-hiMGLs are comparable to CK-hiMGLs

The overexpression of PU.1 during hematopoiesis generated hiHPCs efficiently (Fig. 1). However, we were not sure whether PU.1 could enhance the efficiency of microglia terminal differentiation. Therefore, we next tried to compare the efficiency and purity of hiMGL derivation between the PU and CK protocols.

**Fig. 2:**
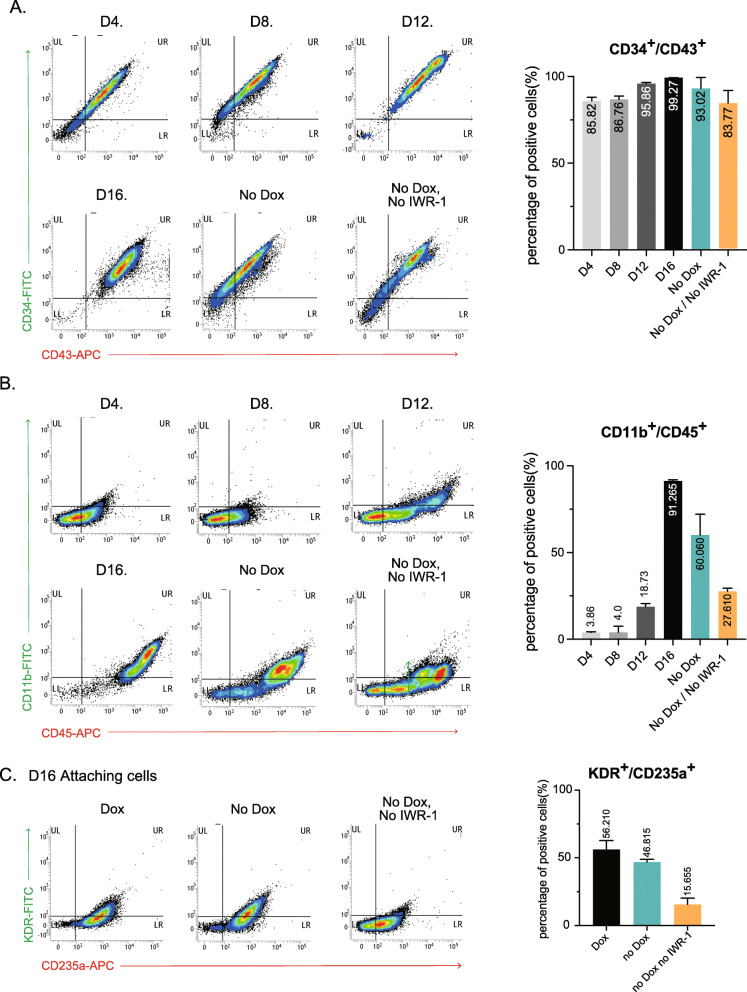
Primitive hematopoietic lineage induction by PU.1 overexpression. **A** Flow cytometry analysis of the differentiation pattern of hiHPCs. Markers of hematopoietic progenitor cells, CD34 and CD43, were analyzed in floating hiHPCs from day 4 to day16 (several thousand cells per independent experiment). **B** Flow cytometry analysis of the differentiation pattern of hiHPCs. Markers of myeloid cells, CD11b and CD45, were analyzed in floating hiHPCs from day 4 to day16 (several thousand cells per independent experiment). **C** Flow cytometry analysis of the differentiation pattern of hemangioblasts. Markers of the primitive hematopoietic lineage, KDR and CD235a, were analyzed in attached mesodermal cells at day16 (several thousand cells per independent experiment)

While the percentage of hiHPCs expressing CD34 and CD43 above 90% were comparable in both methods (Fig. 2A), the percentage of CD11b and CD45-positive cells showed a significant difference between CK and PU protocols (Fig. 2B). In PU protocol, over 90% of the floating hiHPCs (considered to be microglia progenitor cells) were CD11b/CD45-positive on day 16, compared with about 60% in CK protocol, whereas this percentage in PU protocol was about 27% without neither Dox nor IWR-1e treatment (less efficient for primitive hematopoietic progenitors). These results indicated that posterior mesodermal patterning is crucial for the subsequent differentiation of microglial progenitor cells. We further examined the expression of CD235a, a specific marker of the primitive hematopoietic lineage [35], along with a hematopoietic stem cell marker KDR (also known as VEGFR2) in the adherent mesodermal cell population. Notably, we found that without blocking the Wnt/β-catenin pathway, ∼ 16% of mesodermal cells were CD235a/KDR-positive, suggesting that these cell population differentiated into a lineage different from the primitive hematopoietic lineage (Fig. 2C) [35]. In contrast, the appropriate inhibition of canonical Wnt signaling successfully gave rise to ∼ 60% CD235a/KDR-positive mesodermal cells (Fig. 2C, Supplementary Fig. S5, S6). Due to the higher purity and greatly improved yields of microglial progenitor population in PU protocol, we thereafter mainly used PU-hiMGLs, unless otherwise noted.

**Fig. 3:**
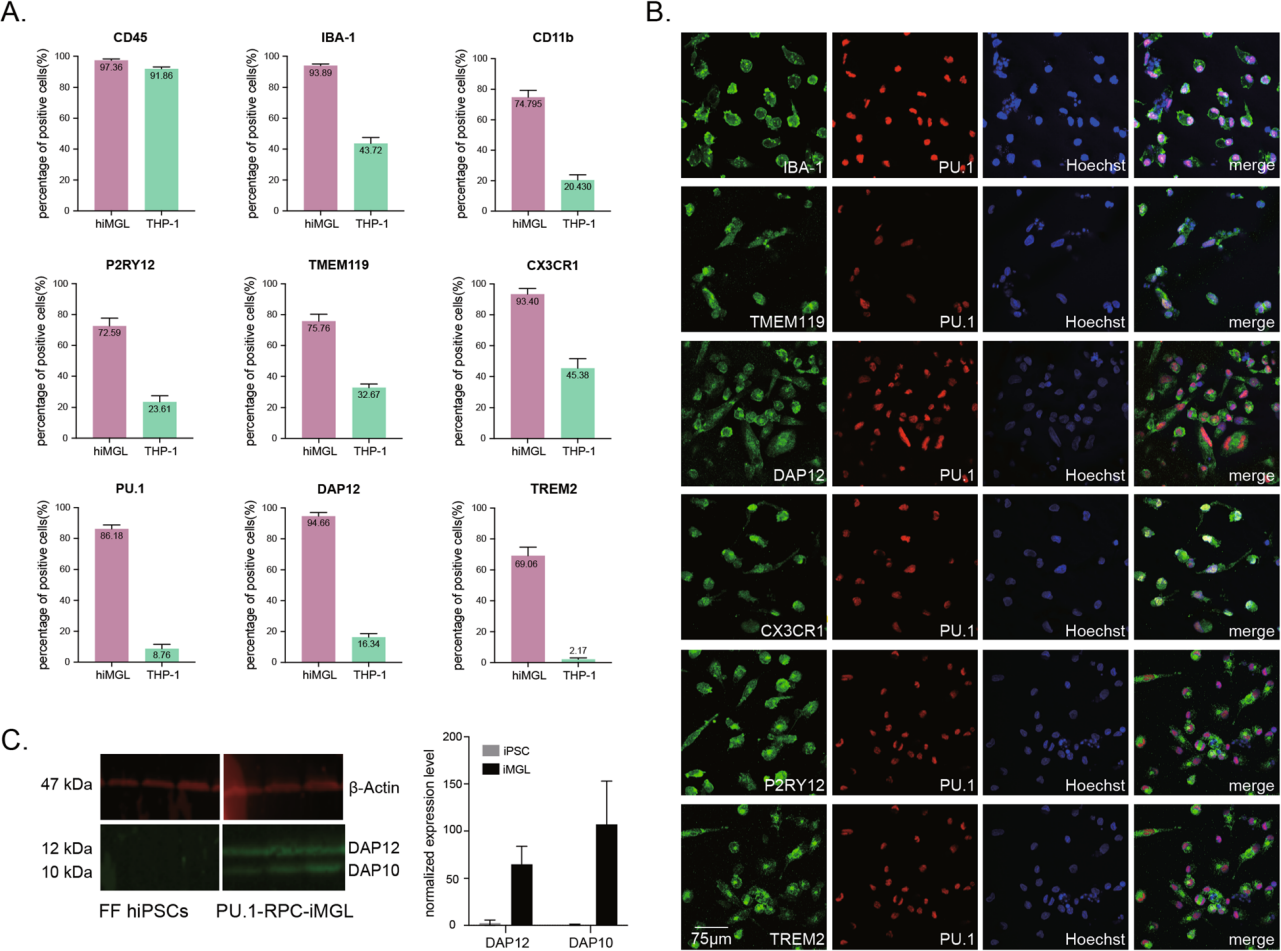
High purity of microglial-like cells in hiMGLs derived by the PU protocol. **A** The purity of iMGLs was analyzed by flow cytometry at day 25 (also see Supplementary Fig 7). Whereas the marker of pan myeloid cells (CD45) was expressed equally by both hiMGLs and monocytic THP-1 population (more than 90%), microglia-specific markers (IBA1, CD11b, P2RY12, TMEM119, CX3CR1, PU.1, DAP12, and TREM2) were enriched in hiMGL compared to THP-1 (several thousand cells per each independent experiment). **B** Immunostaining of microglia-specific markers (IBA1, TMEM119, DAP12, CX3CR1, P2RY12, and TREM2) co-stained with PU.1 and Hoechst 33342 on hiMGLs at day 25. Scale bar, 75μm. **C** Western blot of DAP12 was performed using cell lysate of iPSC and PU-hiMGL. hiPSCs showed a low expression of DAP12 and its highly homologous DAP10 until they were differentiated into iMGLs. Both DAP12 and DAP10 were increased by ∼ 50% and ∼ 100% in cell lysates of hiMGL compared with those of hiPSC (*n* = 3 independent experiments)

We next tested the microglial purity of hiMGLs after hiHPCs were re-plated into culture dishes and cultured for another week. For further comparison with a distinct myeloid population, we used THP-1 cells, a human monocytic cell line, as another myeloid control with different destiny. Although most hiMGLs and THP-1 cells (95%) highly expressed the hematopoietic cell marker CD45, only hiMGLs expressed high levels of myeloid lineage markers (IBA1, PU.1, and CX3CR1), as well as microglia-specific markers (TMEM119 and P2RY12), compared with THP-1 (Fig. 3A). Especially, DAP12 and TREM2, which are specifically expressed in microglial cells, were highly expressed in ∼ 85% and ∼ 95% of hiMGLs, respectively, while almost no THP-1 cells expressed them (Fig. 3A, Supplementary Fig. S7).

Next, we confirmed by immunocytochemistry that most cells re-plated into culture dishes expressed microglia-specific markers, IBA1, TMEM119, CX3CR1, P2RY12, DAP12, and TREM2, with their nuclei co-stained with PU.1 (Fig. 3B). Furthermore, western blotting showed that DAP12 and DAP10 proteins were only expressed in hiMGLs, not in hiPSCs (Fig. 3C). These data implied that the PU protocol could successfully generate hiMGLs specifically expressing microglial markers with high purity in a short period of time.

### PU.1 overexpression does not affect the transcriptional profile of hiMGLs

While PU.1 is a crucial transcription factor for microglia differentiation, a minor variant of PU.1 has been associated with the pathogenesis of AD [39], suggesting that long-term overexpression of PU.1 might result in an alteration of microglia-like cells similar to those in brains of AD patients. Next, we performed a comprehensive gene expression analysis to assess how close hiMGLs related to primary microglia cells collected from a human brain, and whether PU.1 overexpression would affect the characteristics of microglia at transcription levels compared with those in the CK protocol.

**Fig. 4:**
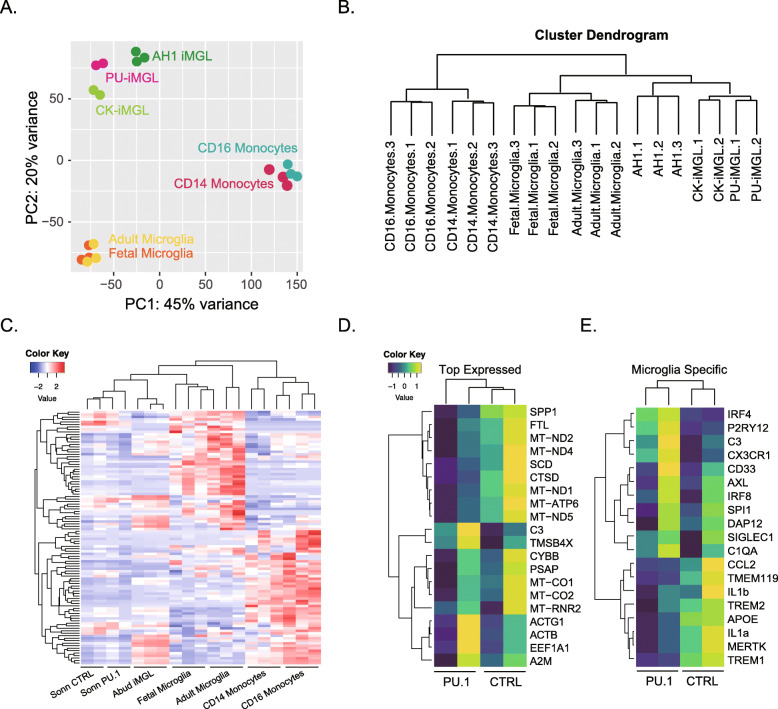
Comparable transcriptome profiles between hiMGL and human primary microglia. **A** Principal component analysis (PCA) of PU-hiMGLs (PU-iMGL, deep pink), CK-hiMGLs (CK-iMGL, chartreuse), human fetal microglia (fetal microglia, chocolate), human adult microglia (adult microglia, gold), human microglia-like cells derived from hiPSCs reported by previous paper (AH1 iMGL, forest green), CD14+/CD16− monocytes (CD14 monocytes, maroon), and CD14−/CD16+ monocytes (CD16 monocytes, deep skyblue) (*n* = 20,651 genes). PCA analysis demonstrated that both hiMGLs derived through the PU and CK protocol were close to human microglia, not to monocytes or macrophages. **B** Hierarchical clustering demonstrated that hiMGLs harvested through both PU and CK protocols were grouped with human microglia cells, instead of monocytes or macrophages. **C** Heatmap and biclustering on 100 genes (FDR < 0.05) most highly expressed by hiMGLs, human microglia, and monocytes. **D** Heatmap of the 20 genes most highly expressed in hiMGLs by the PU or CK protocol. **E** Heatmap of 20 genes selected as representatives of genes exclusively expressed by human microglia. Microglia-specific markers (*P2RY12*, *CX3CR1*) seem to be expressed higher in the PU protocol, while genes linked to inflammatory responses (*IL1a*, *IL1b*, *MERTK*) were highly expressed in iMGLs derived by the CK protocol

Total RNAs of hiMGLs were purified on day 25 in both CK and PU protocols, and their gene expression profiles were compared with monocytes, adult and fetal primary human microglia, and other hiPSC-derived microglia-like cells, which were published previously [16, 17]. RNA sequencing followed by principal component analysis (PCA) and hierarchical clustering analysis visualized by heatmap revealed that the transcriptional profile of hiMGLs was close to those of microglia-like cells derived from another protocol [16] and primary microglia, whereas monocytes showed far different profiles (Fig. 4A–C). These results clearly demonstrated that hiMGLs resembled human primary microglia and could be used as a cellular model for studying human microglia in both physiological and disease conditions.

Furthermore, we analyzed in detail the genes differently expressed in hiMGLs derived from the CK protocol and PU protocols. First, we picked up the genes mostly expressed by hiMGL (Fig. 4D). Worth mentioning, except some housekeeping genes and genes involved in the complement system, *SPP1*, which encodes a protein called osteopontin (OPN), was highly expressed in hiMGLs [38]. Osteopontin was reported to be involved in the polarization, chemotaxis, and phagocytosis functions of immune cells [40]. Furthermore, in addition to the change of microglia features, OPN can also be secreted by microglia, promoting the proliferation of neural precursor cells [41]. Recently, OPN was also reported as a brain region-specific microglial marker, which can affect the phagocytic function of microglia [42–44].

Next, we compared the expression level of various microglia markers between CK- and PU-hiMGLs. Interestingly, PU.1 overexpression seemed to downregulate the expression of inflammatory reaction genes, such as *IRF4*, *IL-1a*, and *C1QA* (Fig. 4E). This impression was further reinforced by the comparison of upregulated and downregulated genes between PU.1 overexpressing cells and their counterparts (Supplementary Fig. S8). Gene Ontology (GO) term enrichment analysis indicated that PU.1 overexpression reduced the expression level of genes involved in response to antigens, such as viruses and bacteria, while genes linked to neurodegenerative diseases were barely affected. This negligible effect of PU protocol on the character of homeostatic microglia is an important observation indicating that this PU protocol can be applied for studying human microglia in the context of neurodegenerative diseases.

### PU-hiMGLs exhibit physiological microglial functions

Microglia sense the alteration of the nearby environment and remove unwanted materials which otherwise would injure brain neural cells [45]. To confirm the physiological function of hiMGLs, we investigated the following microglia-specific features: phagocytosis, secretion of inflammatory cytokines, and formation of the inflammasome.

**Fig. 5:**
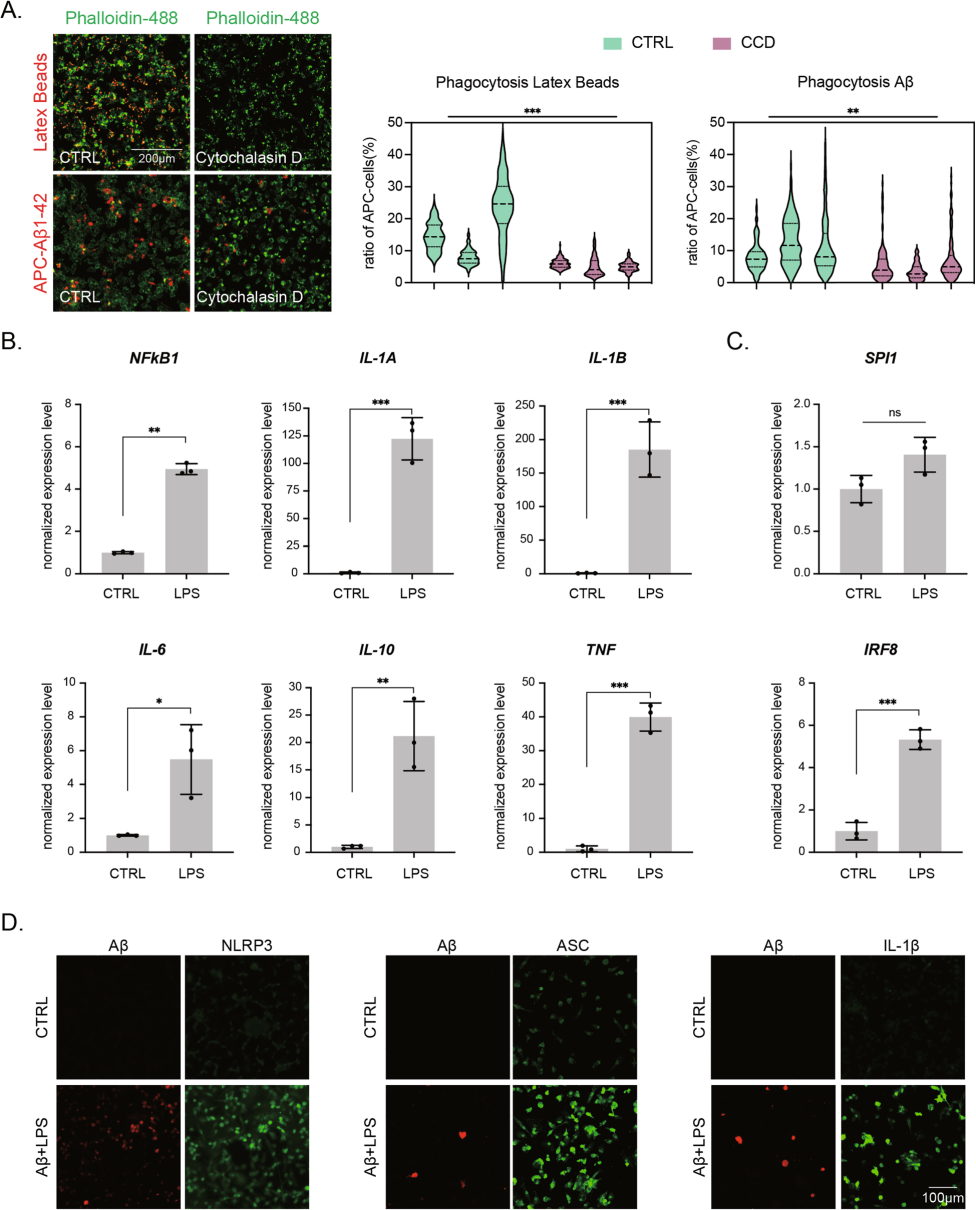
Evaluation of microglial physiological functions in PU-hiMGLs. **A** Phagocytosis analysis using 1.0-μm Latex beads and fibrillar Aβ1–42, both conjugated with APC fluorescence. Representative images of hiMGLs with and without phagocytosis inhibitor, CCD. Scale bar, 200 μm. Quantification of hiMGLs showing phagocytosis of fluorescence. Two-way ANOVA showed a significant difference between the groups with and without CCD. *F* (161, 648) = 4.2, *p* = 0.000835 for Aβ1–42 and *F* (161, 547) = 3.6, *p* = 0.000927 for Latex beads, followed by post hoc test ***p < 0.001, *n* = 3 independent experiments using *n* = 3 independent clones. **B** Levels of cytokine gene expression in iMGLs were quantified by qRT-PCR following 3-h LPS treatment. *p < 0.05; **p < 0.01; ***p < 0.001. All data are expressed as mean ± SEM (*n* = 3 independent clones with *n* = 2 independent experiments). **C** The expression level of two key microglial transcription factors (*SPI1*, *IRF8*) was evaluated by qRT-PCR after 3-h LPS treatment. ns, not significant; *** *p* < 0.001. All data are expressed as mean ± SEM (*n* = 3 independent clones with *n* = 2 independent experiments). **D** Composition of the inflammasome (NLRP3, ASC, IL-1β) was confirmed by immunostaining after LPS and Aβ1–42 co-treatment. Scale bar, 100 μm. SEM, standard error of the mean

Clearance of pathogens or cell debris is one of the most notable functions of microglia, as suggested by the observation that efficient engulfment of fibrillar β-amyloid (Aβ) by microglia is compromised in the brain of AD patients. To confirm the phagocytic ability of hiMGLs, we used 1.0 μm latex beads and fibrillar Aβ1–42, both conjugated with allophycocyanin (APC) fluorescence. Since these particles might attach to the cell surface instead of entering inside the hiMGLs, we used a phagocytosis inhibitor, cytochalasin D (CCD), which prevents actin polymerization. We found that after treatment with CCD for 2 h before the addition of Aβ peptide or latex beads, hiMGLs lost almost all of their phagocytic ability, from ∼ 30% APC-positive cells to ∼ 5% with latex beads and ∼ 10 to ∼ 5% with Aβ peptide, indicating that latex beads and Aβ peptide were successfully endocytosed in this assay condition (Fig. 5A).

Cytokines released by microglia are vital regulatory factors of the innate immune system. Thus, the secretion of inflammatory cytokines by hiMGLs was measured following treatment with lipopolysaccharide (LPS) for 3 h. qRT-PCR showed that the expression of five inflammatory cytokine genes, *IL-1a*, *IL-1β*, *IL-6*, *IL-10*, and *TNF*α, were strongly upregulated by LPS, especially *IL-1β* that was upregulated about 200 times (Fig 5B). Many of these cytokines have been reported to be elevated in the pathological condition AD [46]. Expression of *NFKB1* was also upregulated five times with LPS treatment, indicating the activation of NF-κB signaling (Fig. 5B). Since abnormal NF-κB signaling activation was reported in AD, we believe that these LPS-stimulated hiMGLs might be used for AD research in future studies [47]. Surprisingly, although *SPI1* was not significantly upregulated, *IRF8*, which is another important transcription factor for myeloid cells, was highly elevated following treatment with LPS (Fig. 5C) [48].

Recently, the inflammasome has been reported to be involved in the pathogenesis of two AD-related pathological hallmarks, tau and Aβ [49, 50]. Considering the application of hiMGLs in future studies on AD, we next tested the formation of the inflammasome in hiMGLs. Interestingly, we found that the levels of mRNAs encoding two key proteins of the inflammasome (ASC and NLRP3) and IL-1β were upregulated in hiMGLs after co-treatment with Aβ and LPS (Fig. 5D). However, treatment with LPS alone was sufficient to upregulate the expression of IL-1β, while the increase of inflammasome-related genes (*ASC* and *NLRP3*) was observed with Aβ alone or Aβ/LPS, but not LPS alone (Supplementary Fig. S9, S10). These results confirmed that the hiMGLs were able to form the inflammasome under inflammatory conditions relevant to human primary microglia.

Together, LPS treatment led to NF-κB-dependent upregulation of pro-IL-1β, whereas Aβ treatment alone does not induce the expression of IL-1β. In contrast, inflammasome activation, aggregation of NLRP3 and ASC, was induced fully with Aβ stimulation following the prime signaling such as LP S[51] but was also partially observed with Aβ stimulation alone.

### Development of a novel culture platform containing hiMGLs and primary neurons to mirror the physiological circumstances in the CNS

**Fig. 6:**
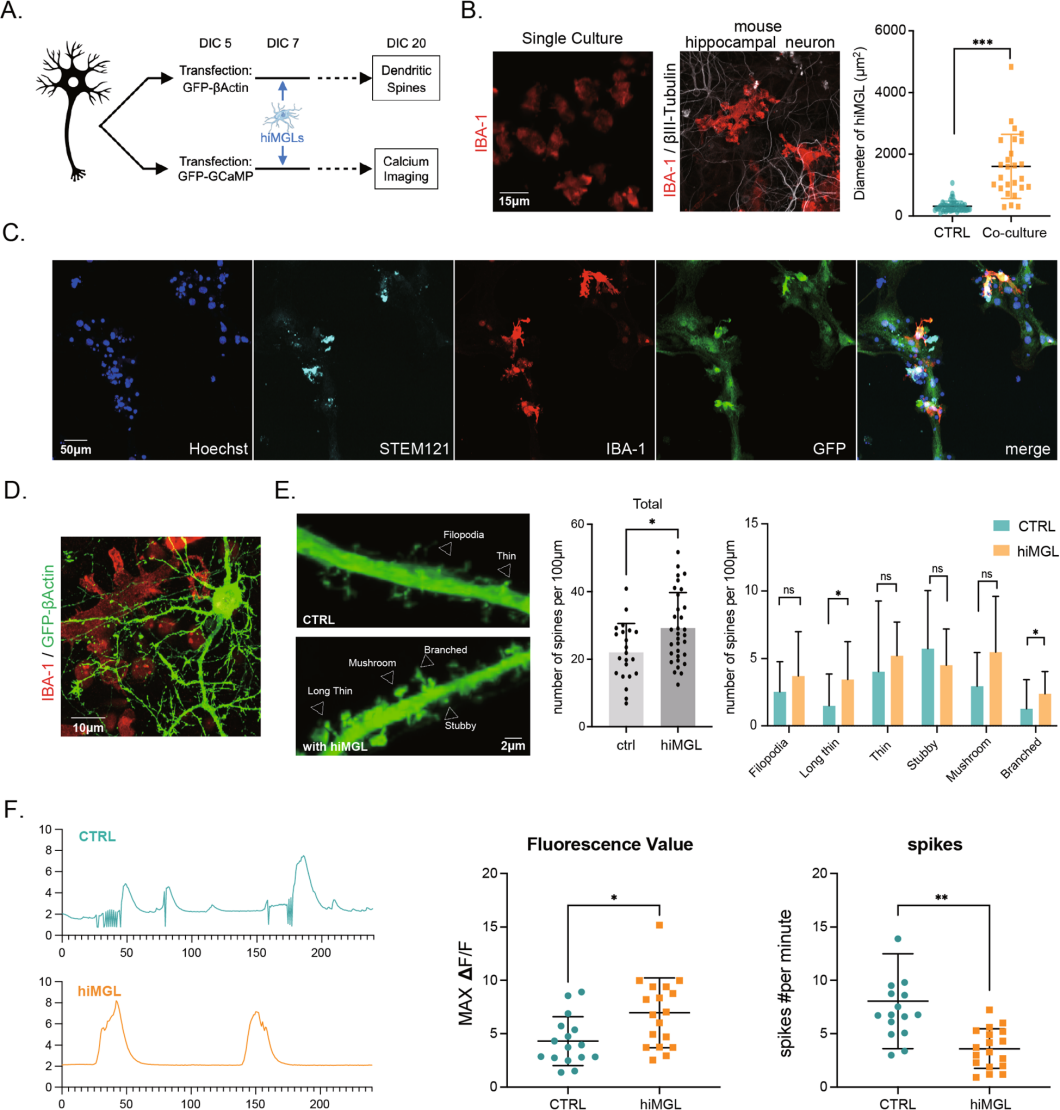
Reciprocal biological effects in co-culture of hiMGLs and primary neurons. **A** Schematic diagram of the co-culture system. **B** Representative images of immunostaining with IBA-1 and βIII-tubulin in co-culture or hiMGLs alone. After co-culture with primary neurons for 1 week, hiMGLs exhibited a more ramified morphology, which was not observed in hiMGL culture alone. In addition, hiMGLs in co-culture showed a larger area than those in single culture (*n* = 32 in single culture, 26 in co-culture; 3 independent experiments). Scale bar = 15μm. ***p < 0.001. **C** Mouse primary neurons were transfected with actin-GFP plasmid and then immunostained with anti-STEM121, IBA1, and GFP antibodies. Scale bar = 50μm. **D** hiMGLs showed attaching to axons and dendrites. Scale bar = 10μm. **E** The number of dendritic spines was increased in co-culture with hiMGLs. High-magnification images showed that neurons co-cultured with hiMGLs had more matured spines (mushroom and so on), while neurons in single culture had much less mature dendritic spines. Scale bar = 2μm. Spines were categorized into six groups and quantified. Quantification showed much more spines in the co-cultures than single cultures (*n* = 25 in single culture, 35 in co-culture; 3 independent experiments). ns, not significant; *p < 0.05. All data are expressed as mean ± SEM. **F** Primary neurons co-cultured with hiMGLs showed large spikes and reduced frequency. Representative images of calcium response in the co-culture (hiMGLs) or neurons alone (CTRL). Quantification of maximum Δ*F*/*F* and spike frequency indicated that hiMGLs could raise the regularity of neuron’s activity (*n* = 16 in single culture, 18 in co-culture; 3 independent experiments). *p < 0.05; **p < 0.01. All data are expressed as mean ± SEM. SEM, standard error of the mean

Impaired microglial functions have been reported in various neurodegenerative diseases, such as AD, ALS, and Parkinson’s disease [52, 53], which affect the neural network in the complex context of the human CNS. To examine if hiMGLs can be applied to more suitable pathophysiological environments, we developed a novel culture platform, where human hiMGLs were co-cultured with mouse primary neurons (Fig. 6A). Because we sought to assess the effect of microglia on functional mature neurons, we used mouse primary neurons instead of human iPSC- or fibroblast-derived neurons, which are well-known for their immature features.

First, to examine the morphological alteration of hiMGLs, we performed immunocytochemistry of the cultures using antibodies against IBA1, a microglia-specific marker. Strikingly, hiMGL morphology changed from ameboid to ramified after co-culture with primary hippocampal neurons for two weeks. In addition, the area of hiMGL cytoplasm was increased by ∼ 10-folds in the co-culture, implying that hiMGLs shifted to a sensing mode (Fig. 6B). Furthermore, a similar morphological alteration of hiMGLs was observed in co-culture with mouse cerebellar neurons (Supplementary Fig. S11a). These results suggested that some neuronal factor(s) could regulate hiMGL features, independently of the brain’s regional origin. Importantly, immunostaining with STEM121, an antibody against human-specific cytoplasm antigen, confirmed that these microglia-like cells in the co-culture system were human-derived, different from mouse primary microglia cells (Fig. 6C).

In early development, microglia play a key role in pruning supernumerary synapses and dendritic spines [54, 55]. Recently, microglia have been reported to control stimulus-dependent spine structural changes at synapses in adult mouse brains [56, 57]. To further explore how hiMGLs affect neuronal morphology and function, we analyzed dendritic spines and neuronal activity. For clearer effects of hiMGLs, we used mouse primary neurons, as human microglia can be successfully transplanted into mouse brains without any developmental disturbance albeit species difference [58]. For the analysis of dendritic spines, we transfected mouse primary neurons with a plasmid expressing sparsely β-actin-GFP fusion protein to visualize spine morphology in the cultures (Fig. 6D). Then, we quantified the dendritic spines classified into six groups: filopodia, long thin, thin, stubby, mushroom, and branched. Surprisingly, the number of spines in primary hippocampal neurons was significantly increased by ∼ 1.5-folds in co-culture with hiMGLs, which was mostly contributed by spines with long thin and branched morphology (Fig. 6E). Although not reaching statistical significance, the number of mushroom spines showed a tendency to increase in co-culture with hiMGLs, indicating that hiMGLs could promote the maturation of dendritic spines in mouse primary hippocampal neurons (Fig. 6D, E). Unexpectedly, however, we were unable to find significant effects of hiMGLs on mouse cerebellum neurons (Supplementary Fig. S11). This discrepancy could be due to different functional characteristics of microglia regarding distinct regions of the brain. In agreement with such an idea, microglia show different densities and characteristics in different brain regions, although these cellular and molecular particularities remain to be elucidated [44, 59]. To evaluate the spontaneous neuronal activity, we transfected mouse primary neurons with a plasmid expressing the fluorescent calcium indicator GCaMP6s under the neuron-specific *Synapsin I* promoter. Neurons co-cultured with hiMGLs showed a reduced spike frequency and a higher maximum activity than those cultured in the absence of hiMGLs (Fig. 6F), suggesting that besides structural alteration of spines, the neuronal activity is modulated by hiMGLs.

In summary, we demonstrated that hiMGLs could be used to investigate physiological neuronal functions through a novel in vitro system, which comprised primary neurons and hiMGLs. This platform could be applied to study neuron-microglia interactions and pathophysiological mechanisms when co-culturing with patient-derived cells carrying relevant gene mutations.

## Discussion

Since Pío del Río-Hortega described oligodendrocytes and microglia together as the “third element” of CNS in 1919, over a century has passed. While the microglial cellular origin remained elusive for a long time, its ontogeny has been convincingly demonstrated by recent findings pointing to the yolk sac, which originates in the extra-embryonic mesoderm, in contrast to the other cellular neural elements that arise from the neuroectoderm [18]. However, as most studies were accomplished using postmortem specimens or non-human materials thus far, there is an urgent and strong need to develop a new method for providing a sufficient number of human microglia. In the present study, in relation to the cellular ontogeny of microglia, we developed a novel method to derive microglial cells from hiPSCs faster and more efficiently than with previously established protocols. Especially, to drive cellular ontogeny into primitive hematopoietic cells, we used small chemicals regulating the Wnt pathway at appropriate time points. By forcing the expression of the transcription factor, PU.1, we successfully harvested a large amount of microglia within three weeks. Transcriptome analyses demonstrated that our hiMGLs were transcriptionally very close to human primary microglia (Fig. 4). Even without cell sorting, the purity of these hiMGLs was over 90%, which was confirmed by both flow cytometry and immunostaining (Fig. 3). Regarding cellular functions, hiMGLs could phagocyte latex beads and Aβ peptide, which were further confirmed by the phagocytosis inhibitor, CCD (Fig. 5A). Major upregulation of inflammatory cytokine expression in hiMGLs was observed after 3-h LPS treatment (Fig. 5B), while the incubation with Aβ peptide and LPS also induced the inflammasome (Fig. 5D). Furthermore, in the co-culture model, calcium imaging showed that mouse primary neurons co-cultured with hiMGLs had more dendritic spines and stronger regular spikes (Fig. 6), indicating that hiMGLs helped neurons to maturate and form appropriate functional networks.

Only a few methods have been reported for deriving microglia-like cells from human iPSCs since the first report in 2016 [14–17], and most of them demand a relatively long culture period (about two months) combined with cell sorting by flow cytometry.

In contrast, we showed in this study that human microglia cells could be generated from hiPSCs in an efficient and simple way through overexpression of PU.1, a vital transcription factor for microglia development, without cell sorting and any feeder cells. By using this more efficient and economic protocol, a large number of microglia, enough for RNA extraction or protein purification, could be harvested even from a small number of starting hiPSCs (approximately 120-folds), compared with other protocols (at most ~ 40 folds). hiMGLs could be derived within 3 weeks and maintained over a long duration (> 2 months), whereas other protocols sometimes took ~ 60 days. In addition to the known association of PU.1 with inflammatory responses, genome-wide association studies have shown that the expression levels of PU.1 contribute to late-onset AD [60, 61]. Therefore, we were worried that the transient overexpression of PU.1 would cause an inflammatory activation in hiMGLs. However, the transcriptome profile indicated that PU-hiMGLs have lower expression levels of genes related to inflammation than CK-hiMGLs. Importantly, we have confirmed that hiMGLs could be applied to various functional analyses of microglial physiology.

To our annoyance, the ontogeny of microglia and macrophages resembles each other in their early development. Especially, PU.1 is reportedly crucial for the differentiation of both cell types [21, 62]. Chen et al. recently developed a similar method to derive microglia-like cells from iPSCs by transduction of both PU.1 and C/EBPα [63]. Interestingly, these master regulators have been used for trans-differentiation of macrophage-like cells from human lymphocytes and fibroblasts [64, 65]. Considering that microglia and macrophages were derived from primitive and definitive hematopoietic progenitors, respectively, our strategy had a great advantage for microglial-like cells in that overexpression of PU.1 was initiated after the induction of primitive hematopoietic progenitors. In contrast to our study, Chen et al. transduced two transcription factors (PU.1 and C/EBPα) into iPSCs by lentiviruses. Considering that viral infection might cause an immune reaction, non-viral transduction methods, especially via inducible expression by Dox treatment, could be preferable as shown in this study. hiPSC clones bearing *SPI1* expression plasmid could also be stored as usual to reduce the variations among batches. Another important difference between Chen et al.’s method and ours is the number of transcription factors manipulated and the timing of induction during derivation: both *SPI1* and *CEBPA* were ectopically expressed in hiPSCs in Chen et al.’s strategy, while only *SPI1* was induced in the posterior mesoderm in our study, which we thought might lead to a much larger number of microglia progenitor cells that could be harvested. Analyzed by flow cytometry, our hiMGLs showed a higher expression of CD11b and CX3CR1. Furthermore, although treated only with LPS instead of LPS and IFN-γ, the expression of IL-10 was upregulated in hiMGLs, which contrasted with Chen et al.’s data. Providing that IL-10 is reported to be an anti-inflammatory cytokine, we hypothesized that this was related to our transcriptome data, in which PU.1 overexpression led to lower expression of genes related to inflammation (Fig. 4).

Microglia have been reported to affect neuronal morphology and function in vivo and in vitro [66, 67]. In this study, to understand these microglia-neuron physiological interactions, we have established the experimental system by co-culturing hiMGLs and primary neurons. Here, we showed that hiMGLs exhibited a strikingly more ramified and complex morphology in this co-culture system than in culture alone. Conversely, hiMGLs also changed the characteristics and activity of primary hippocampal neurons, although the detailed molecular mechanism remained to be determined. In a previous study, when microglia were depleted by PLX5662, a CSF1R inhibitor, neurons had fewer responses to odors, indicating that they were not properly connected to neural circuits [5]. Furthermore, in the mice treated with PLX5662, neurons had a lower spine density. In our co-cultured system with hiMGLs, primary neurons exhibited more dendritic spines, suggesting that microglia could help form new synapses or stabilize the existing spines. Indeed, recent studies have shown that microglia influence synaptic formation and maturation during development by direct contact and by releasing factors such as fractalkine, IL-10, and BDNF [56, 67–69]. In this regard, the contradictory effect of hiMGLs on spine density between the hippocampus (Fig. 6E) and cerebellum (Supplementary Fig. S11) might result from a possible different expression of receptors between these neurons such as IL-10 receptor [40, 69, 70]. In addition to the change of spine morphology, we found a significant alteration of neuronal activity in primary neurons with hiMGLs as measured by the calcium indicator (Fig. 6F). In fact, recent studies have demonstrated a reciprocal relationship between microglia and neurons in vivo [66]. It appears that hiMGLs can tune neuronal activity appropriately as primary microglia do. Considering the important role of microglia on synaptogenesis, these results strongly suggest that this co-culture system will prove useful for studying the interactions between neurons and microglia and investigate the pathophysiological mechanisms involving microglia in various neurodegenerative diseases.

The hiMGLs in this study will apply to several neural disease models in the future. While disease conditions, such as AD, would disrupt and dysregulate the defensive function of microglia [52], what intrigued most scientists’ interests was that multiple genes that were specifically expressed in microglia have been identified as pathogenic clues or risk genes in various neurodegenerative diseases, especially *TREM2* in AD [71–73]. Recently, by the beneficial usage of novel methodologies such as transcriptome analysis by single-cell RNA sequencing and high-resolution imaging technology, an understanding of the physiologic function of subpopulations of microglia, as well as microglia in neurodegenerative diseases has tremendously progressed [43, 74, 75]. Here, we demonstrated that the inflammasome could be formed in microglia in response to LPS and Aβ peptide co-stimulation. As previously reported, the inflammasome is involved in amyloid and tau pathology in the brain of AD patients [49]. Coincidentally, although there has been a great deal of studies about TREM2 in AD, the pathogenic mechanism is still unclear.

Overall, our newly developed method for deriving human microglia cells can potentially be applied to transplantation into brain organoids or animal brains to create more relevant experimental models for disease research or for studying the physiology of microglia. Through transcriptome analysis, we also detected that HLA-A was expressed by both CK-hiMGLs and PU-hiMGLs (Supplementary Table 4). Since hiMGLs might have the potential of transplantation for the treatment of various diseases, we think that it is crucial to analyze the expression pattern of other HLA genes in the future [76]. This technology would accelerate the research to recapitulate the in vivo signatures of human microglia.

## Conclusions

By enforcing the expression of a single critical transcription factor, PU.1, we developed a novel method for generating a large number of microglia from hiPSCs in a short period. The resulting hiPSC-derived microglia exhibited normal microglial functions such as phagocytosis and inflammatory responses. This newly developed protocol will pave the way for further studies of human microglia in both physiological and disease conditions.

## Supplementary Information

**Additional file 1: Fig. S1.** BMP4 signaling is crucial for hematopoietic stem cells development. (A) Schematic diagram of mesoderm development in vivo. (B) Immunostaining confirmed that both BMP4 and Wnt signaling were necessary for mesoderm formation in the first two days during differentiation. (C) Images indicating that either VEGF, activating or inhibiting TGF-β1 signaling could stimulate mesoderm formation. (D) The expression level of mesoderm markers was tested by qRT-PCR, confirming neither Activin A nor VEGF would enhance mesoderm formation, while Activin A might be able to inhibit the formation of paraxial mesoderm, opposite to posterior mesoderm. **Fig. S2.** Appropriate inhibition of Wnt signaling is crucial for primitive hematopoietic progenitor cell development. * *p* < 0.05; ** *p* < 0.01; *** *p* < 0.001. All data are expressed as mean ± SEM (n = 3 independent clones with n=3 independent experiments). **Fig. S3.** *SPI1* expression levels in CK- and PU-protocols. (A) *SPI1* and *IRF8* expression patterns in CK-protocol from day 6 to day 18 were quantified by qRT-PCR (n = 3 independent experiments). (B) *SPI1* was upregulated in other iPSC lines (201B7 and WD39) on day 7 in PU-protocol. This increase was confirmed by the expression level of the congruent transcript *βGeo*, while the 3’UTR of *SPI1*, which is not included in *SPI1* expression plasmid, was not changed, indicating that the increased *SPI1* was of exogenous origin. **Fig. S4.** Representative images during iMGLs differentiation. Bubble-like structure that formed in the later stage of the PU-protocol (A) was not observed in the CK-protocol (B). In the last image, scale bar = 75 μm, scale bar = 200 μm otherwise. **Fig. S5.** The PU-protocol can induce more myeloid cells. The ratios of HPCs that are positive for myeloid cell markers, MHCII and F4/80, were determined by flow cytometry. On day 16, the MHCII+/F4/80+ cell population was increased by more than 25% in the PU-protocol (A) compared with the CK-protocol (B). Color curves in histogram indicated isotype control. **Fig. S6.** PU-protocol has a stronger ability to induce cells to the primitive hematopoietic lineage. (Related to Fig. 3 and **Supplementary Fig. 5**) Ratios of positive cells of hematopoietic progenitor cell markers (A, B: CD43 and CD34) and myeloid cell markers (C, D: CD11b and CD45) were analyzed by flow cytometry. The ratio was not changed significantly between PU-protocol (A, C) and CK-protocol (B, D). Color curves in histogram indicated isotype control. **Fig. S7.** Purity of hiMGLs analyzed by flow cytometry. After re-plated on culture dishes for one week, hiMGLs were stained with microglia-specific markers or myeloid cell markers, then analyzed by flow cytometry. A human monocyte cell line, THP-1 cells, was used as a comparative cell population to confirm the potency of antibodies. Color curves in histogram indicated isotype control. (A) The myeloid lineage marker CD45 was equally expressed in hiMGLs and THP-1. (B-F) Microglia specific markers, IBA1 (B), CD11b (C), P2RY12 (D), TMEM119 (E), and CX3CR1 (F) were expressed by all hiMGLs cells, while only a small population of THP-1 cells expressed them. (G) The major transcription factor of microglia, PU.1, was expressed by most hiMGLs cells, while almost none THP-1 cells expressed PU.1. (H, I) The AD risk gene products, DAP12 and TREM2, were expressed by most hiMGLs, while few THP-1 cells were positive for both proteins. **Fig. S8.** Gene expression level modestly varied between CK- and PU-protocol. (A) Heatmap showing the top 20 genes that were upregulated by PU.1 overexpression. (B) GO term enrichment analysis indicated that the genes upregulated by PU.1 overexpression were mostly related to pathogen response. (C) Heatmap showing the top 20 genes that were downregulated by PU.1 overexpression. (D) GO term enrichment analysis indicated that genes downregulated by PU.1 overexpression were mostly related to inflammation. Together with (B), the transcriptome profile showed that PU.1 overexpression would not alter the characteristics of microglia in the central nervous system. **Fig. S9.** hiMGLs were able to form the inflammasome in response to LPS or Aβ peptide stimulation. Immunostaining images demonstrated that IL-1β expression was induced by LPS treatment, but not Aβ peptide. Scale bar = 20 μm. **Fig. S10.** Aβ peptide, but not LPS alone, was able to stimulate the formation of inflammasome. (Related to Fig. 5D, **Supplementary Fig. 9**) Although LPS alone was able to stimulate the expression of IL-1β, inflammasome formation was not stimulated by LPS alone. While LPS treatment followed by incubation together with Aβ peptide has induced the expression of NLRP3 and ASC, Aβ peptide alone was also able to upregulate the expression of NLRP3 and ASC, despite weaker expression without pre-treatment of LPS. Scale bar = 100 μm. **Fig. S11.** No significant differences in dendritic spines were detected between primary neurons from mouse cerebellum co-cultured with hiMGLs or in monoculture. (A) hiMGLs showed a more ramified morphology after co-culture with cerebellar neurons. Scale bar = 5 μm. (B) Primary granule neurons were transfected with β-actin -GFP plasmid. This morphological difference was not observed with or without hiMGL co-culture. Scale bar = 30 μm. (C) There was no significant difference in the number of spines between the single culture and the co-culture system. (n = 8 in single culture, 27 in co-culture; 3 independent experiments). ns, not significant. All data are expressed as mean ± SEM. (D) Spines were counted according to different morphological groups. No significant differences were detected.

**Additional file 2: Supplementary Table S1.**

**Additional file 3: Supplementary Table S2.**

**Additional file 4: Supplementary Table S3.**

**Additional file 5: Supplementary Table S4.**

## Data Availability

RNA sequencing data have been deposited in the Gene Expression Omnibus of NCBI (https://www.ncbi.nlm.nih.gov/geo/) under accession no. GSE178284.

## Abbreviations

iPSCs: Induced pluripotent stem cells
hiMGLs: Microglia induced from hiPSCs
hiHPCs: Hematopoietic progenitor cells induced from hiPSCs
CK protocol: Protocol used to generate hiMGLs by multiple cytokines
PU protocol: Protocol used to generate hiMGLs by forcing the overexpression of *SPI1* as well as application of multiple cytokines
DOX: Doxycycline
OPCs: Oligodendrocyte progenitor cells
SVZ: Subventricular zone
CHIR: CHIR99021
SB: SB431542
PDL: Poly-d-lysine
OPN: Osteopontin
NHD: Nasu-Hakola disease
Aβ: Amyloid beta
CCD: Cytochalasin D
LPS: Lipopolysaccharide
ASD: Autism spectrum disorder
AD: Alzheimer’s disease
FTD: Frontotemporal dementia
PD: Parkinson’s disease
HD: Huntington’s disease
