# Supplementary material for: Single transcription factor efficiently leads human induced pluripotent stem cells to functional microglia
Source: Inflamm Regen. 2022 Jul 1;42:20. doi: 10.1186/s41232-022-00201-1 (PMC9248164; doi:10.1186/s41232-022-00201-1)
Supplement: Supplementary file 1 — Additional file 1: Fig. S1. BMP4 signaling is crucial for hematopoietic stem cells development. (A) Schematic diagram of mesoderm development in vivo. (B) Immunostaining confirmed that both BMP4 and Wnt signaling were necessary for mesoderm formation in the first two days during differentiation. (C) Images indicating that either VEGF, activating or inhibiting TGF-β1 signaling could stimulate mesoderm formation. (D) The expression level of mesoderm markers was tested by qRT-PCR, confirming neither Activin A nor VEGF would enhance mesoderm formation, while Activin A might be able to inhibit the formation of paraxial mesoderm, opposite to posterior mesoderm. Fig. S2. Appropriate inhibition of Wnt signaling is crucial for primitive hematopoietic progenitor cell development. * p < 0.05; ** p < 0.01; *** p < 0.001. All data are expressed as mean ± SEM (n = 3 independent clones with n=3 independent experiments). Fig. S3. SPI1 expression levels in CK- and PU-protocols. (A) SPI1 and IRF8 expression patterns in CK-protocol from day 6 to day 18 were quantified by qRT-PCR (n = 3 independent experiments). (B) SPI1 was upregulated in other iPSC lines (201B7 and WD39) on day 7 in PU-protocol. This increase was confirmed by the expression level of the congruent transcript βGeo, while the 3’UTR of SPI1, which is not included in SPI1 expression plasmid, was not changed, indicating that the increased SPI1 was of exogenous origin. Fig. S4. Representative images during iMGLs differentiation. Bubble-like structure that formed in the later stage of the PU-protocol (A) was not observed in the CK-protocol (B). In the last image, scale bar = 75 μm, scale bar = 200 μm otherwise. Fig. S5. The PU-protocol can induce more myeloid cells. The ratios of HPCs that are positive for myeloid cell markers, MHCII and F4/80, were determined by flow cytometry. On day 16, the MHCII+/F4/80+ cell population was increased by more than 25% in the PU-protocol (A) compared with the CK-protocol (B). Color curves i [file 41232_2022_201_MOESM1_ESM.pdf]

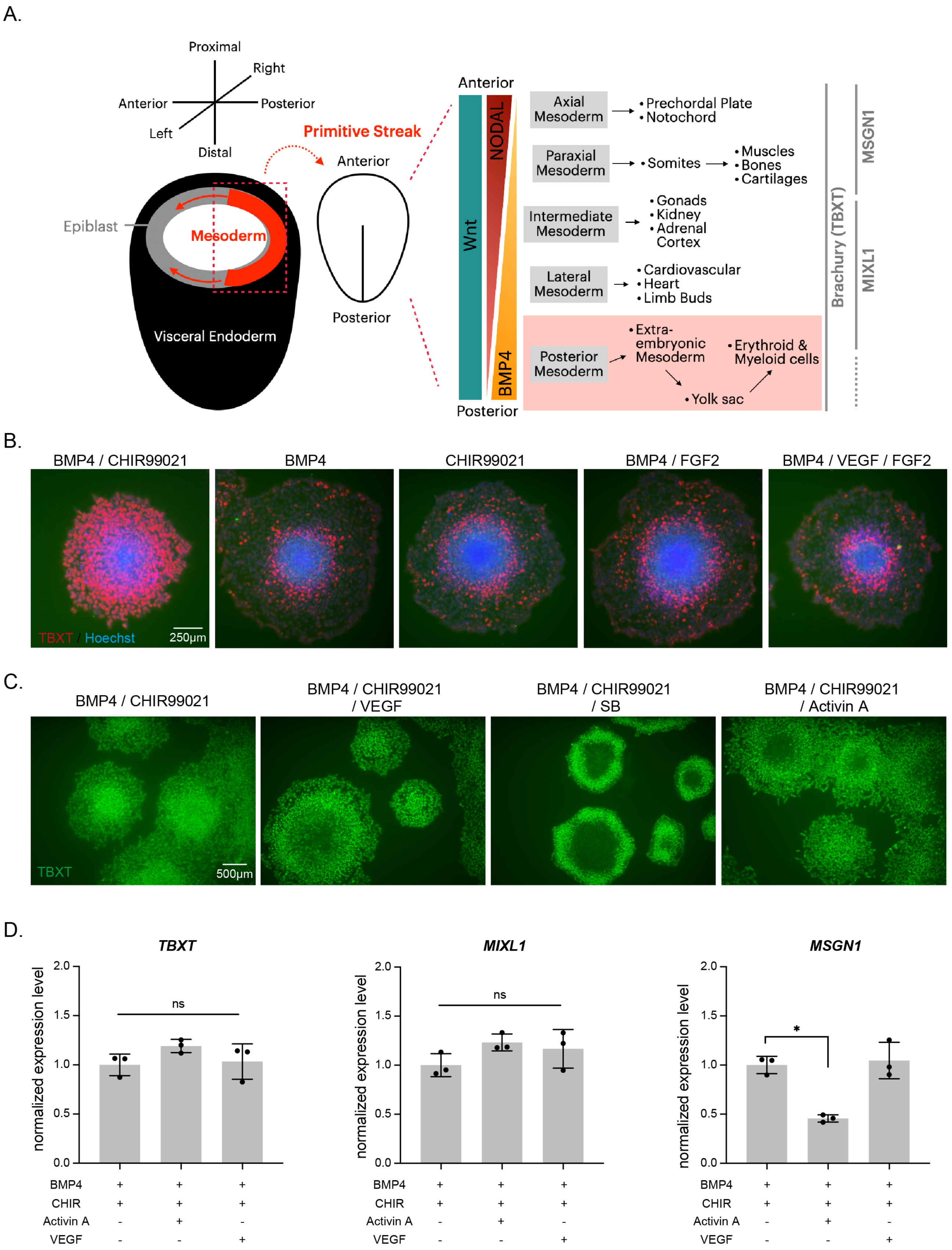

Supplementary Fig. 1 BMP4 signaling is crucial for hematopoietic stem cells development.

(A) Schematic diagram of mesoderm development *in vivo*.

(B) Immunostaining confirmed that both BMP4 and Wnt signaling were necessary for mesoderm formation in the first two days during differentiation.

(C) Images indicating that either VEGF, activating or inhibiting TGF-β1 signaling could stimulate mesoderm formation.

(D) The expression level of mesoderm markers was tested by qRT-PCR, confirming neither Activin A nor VEGF would enhance mesoderm formation, while Activin A might be able to inhibit the formation of paraxial mesoderm, opposite to posterior mesoderm.

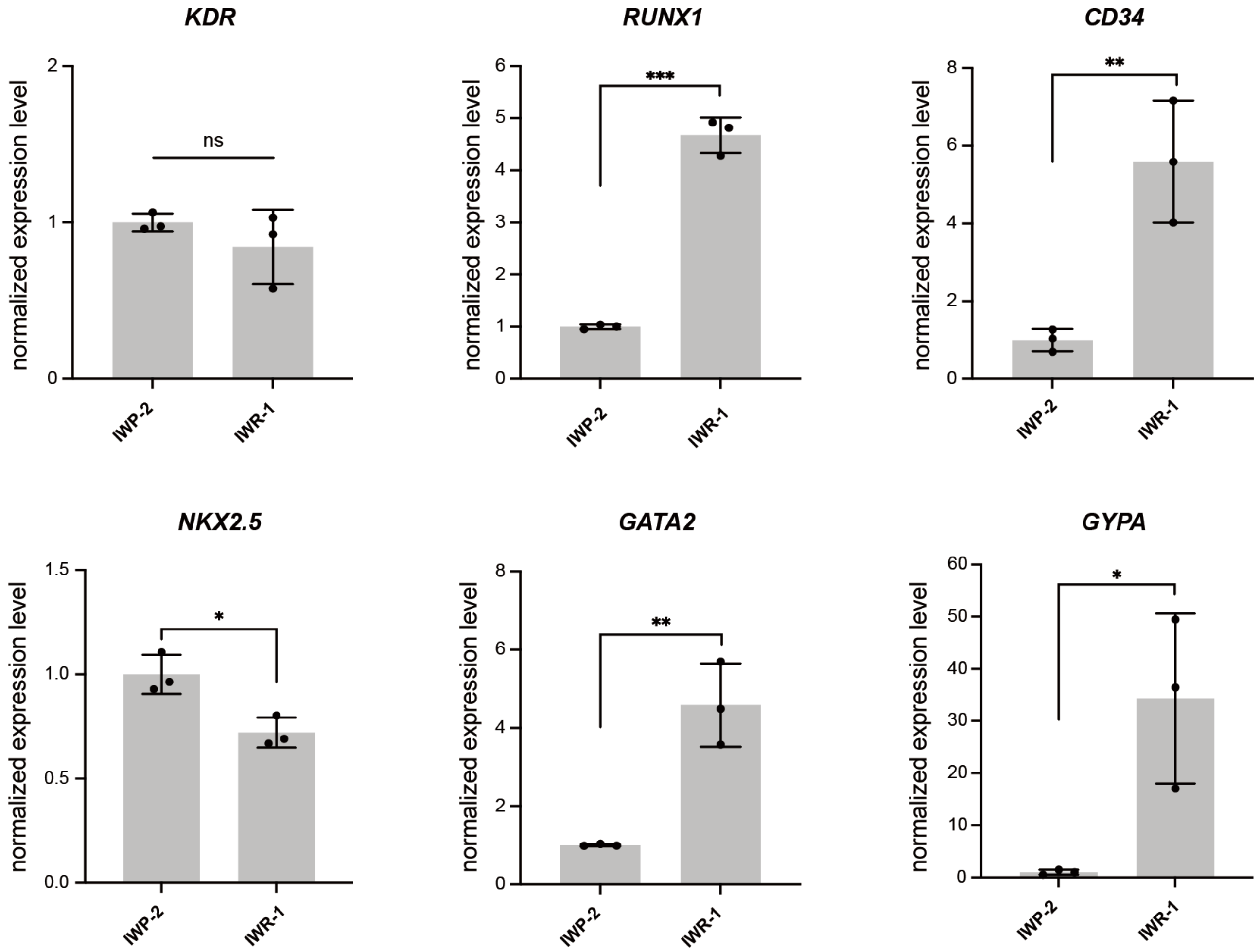

Supplementary Fig. 2 Appropriate inhibition of Wnt signaling is crucial for primitive hematopoietic progenitor cell development.

* $p < 0.05$; ** $p < 0.01$; *** $p < 0.001$. All data are expressed as mean ± SEM (n = 3 independent clones with n=3 independent experiments).

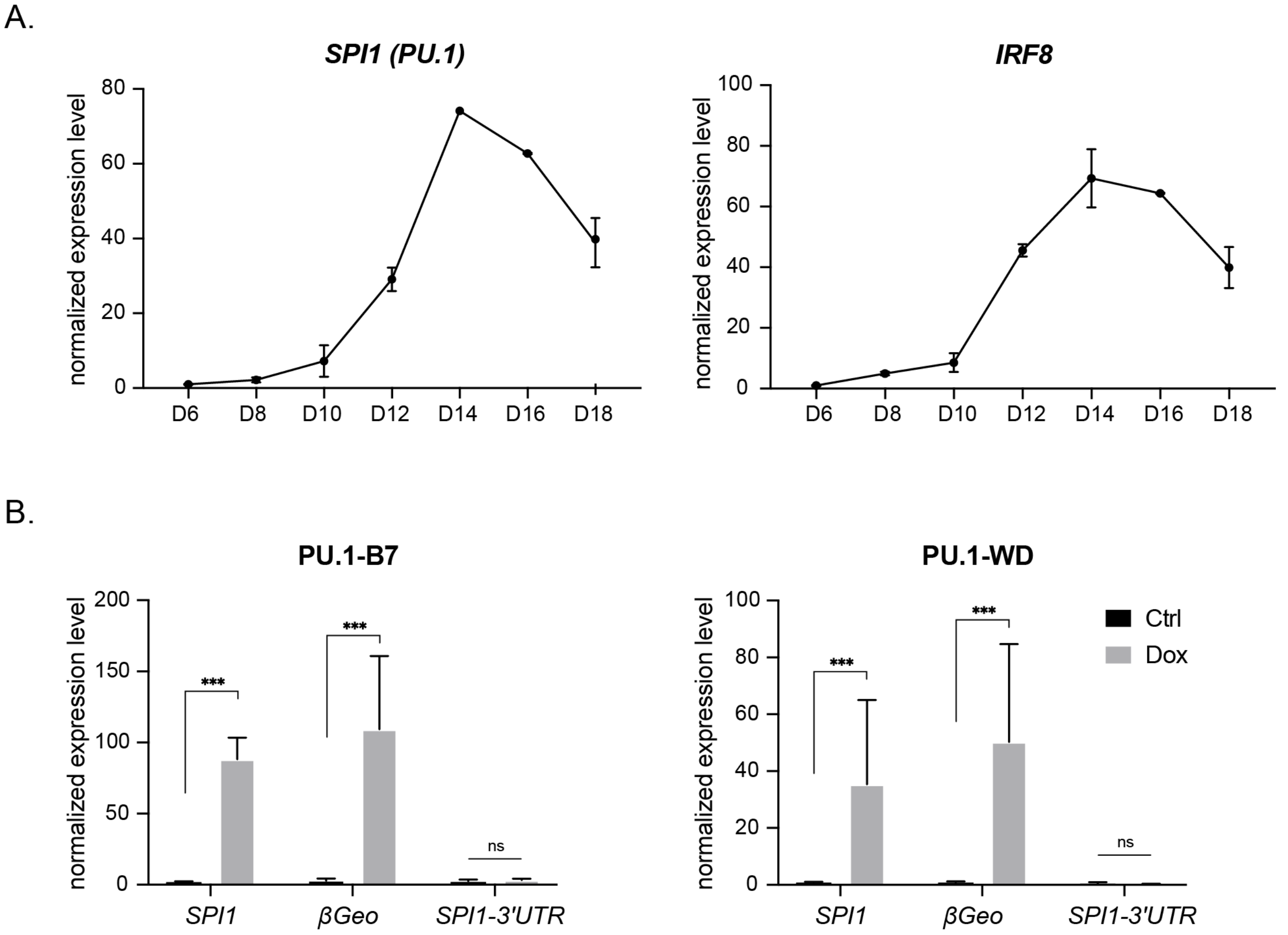

Supplementary Fig. 3 *SPI1* expression levels in CK- and PU-protocols.

(A) *SPI1* and *IRF8* expression patterns in CK-protocol from day 6 to day 18 were quantified by qRT-PCR (n = 3 independent experiments).

(B) *SPI1* was upregulated in other iPSC lines (201B7 and WD39) on day 7 in PU-protocol. This increase was confirmed by the expression level of the congruent transcript *βGeo*, while the 3'UTR of *SPI1*, which is not included in *SPI1* expression plasmid, was not changed, indicating that the increased *SPI1* was of exogenous origin.

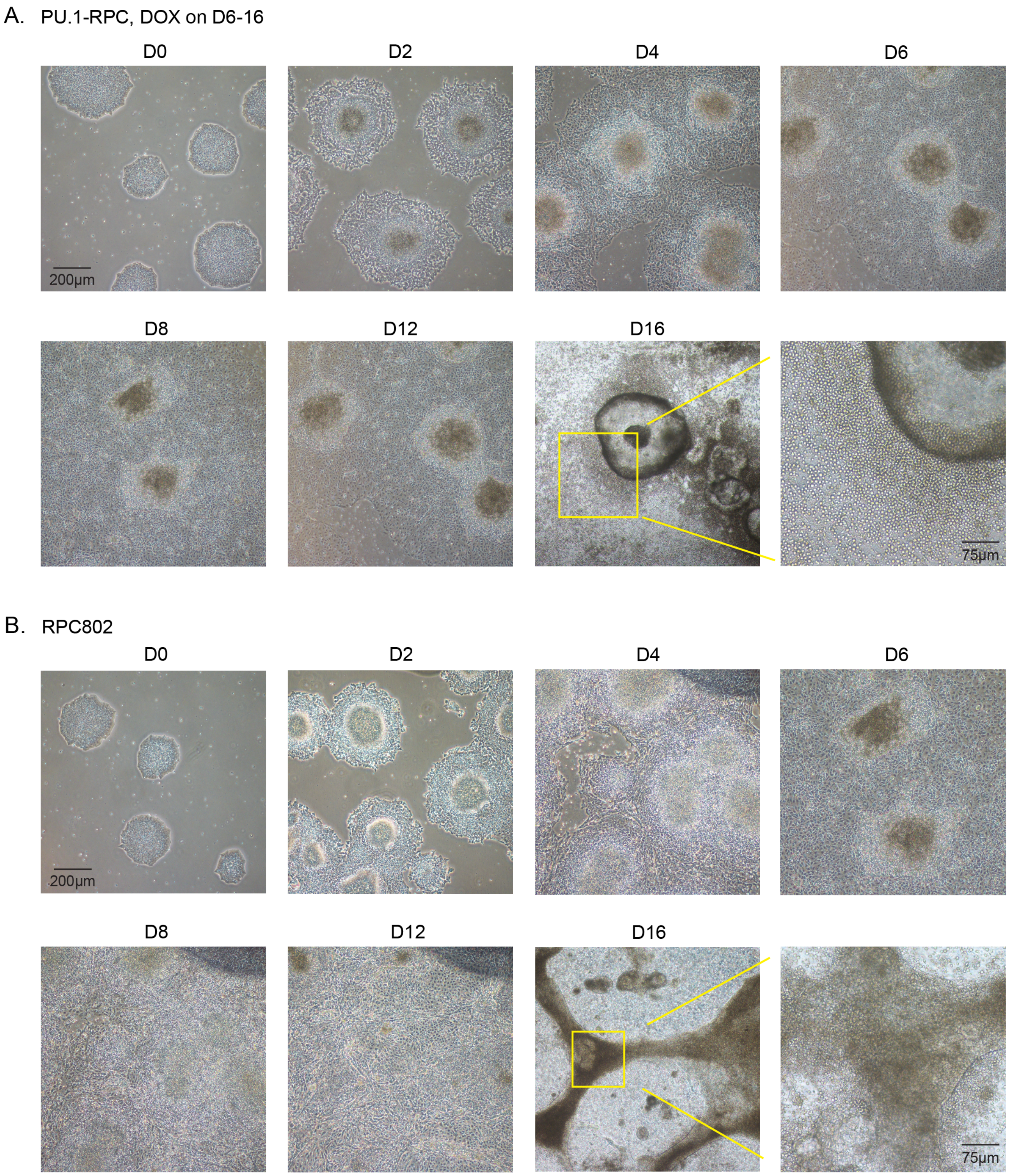

Supplementary Fig.4 Representative images during iMGLs differentiation. Bubble-like structure that formed in the later stage of the PU-protocol (A) was not observed in the CK-protocol (B). In the last image, scale bar = 75 µm, scale bar = 200 µm otherwise.

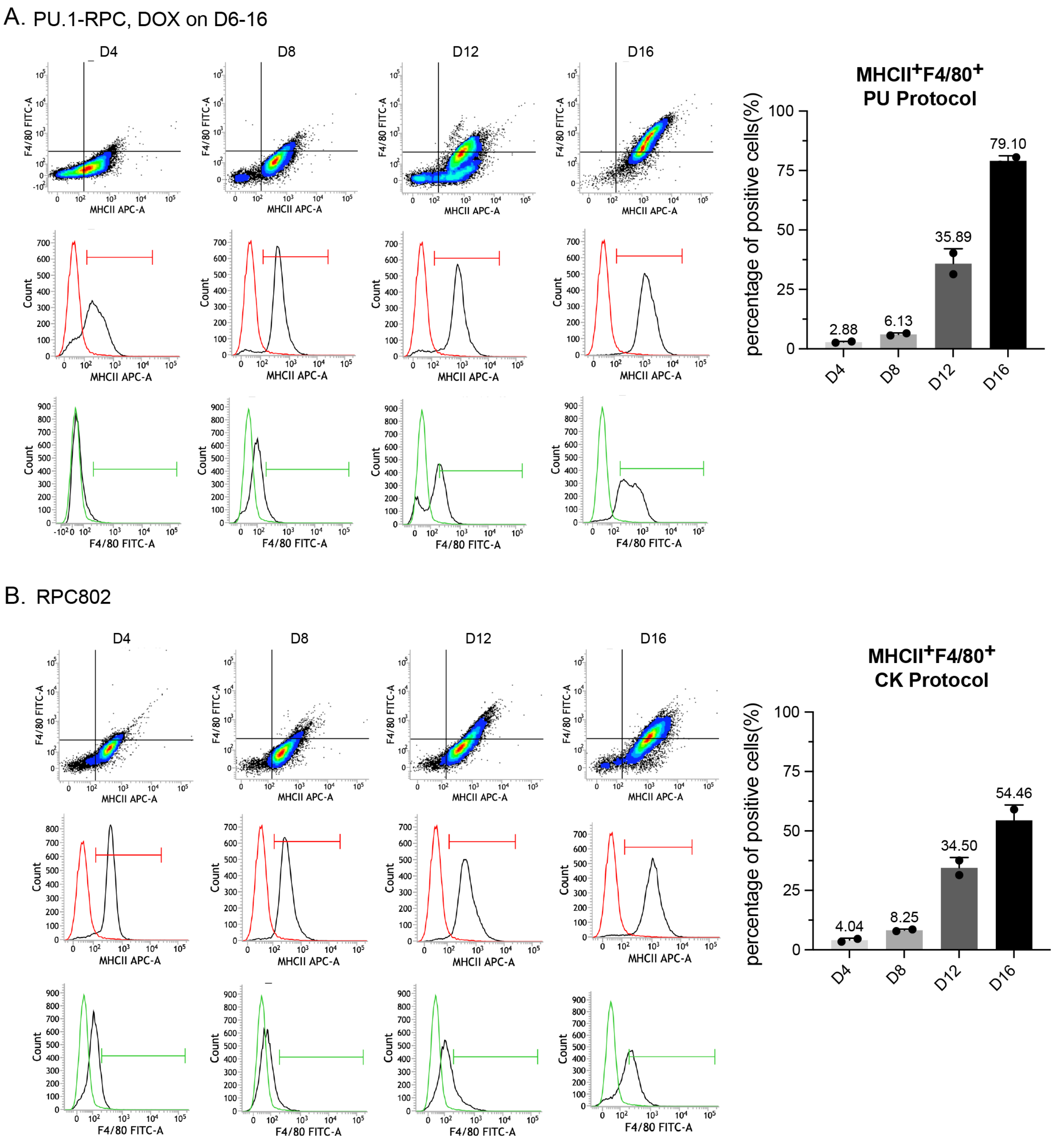

Supplementary Fig. 5 The PU-protocol can induce more myeloid cells. The ratios of HPCs that are positive for myeloid cell markers, MHCII and F4/80, were determined by flow cytometry. On day 16, the MHCII+/F4/80+ cell population was increased by more than 25% in the PU-protocol (A) compared with the CK-protocol (B). Color curves in histogram indicated isotype control.

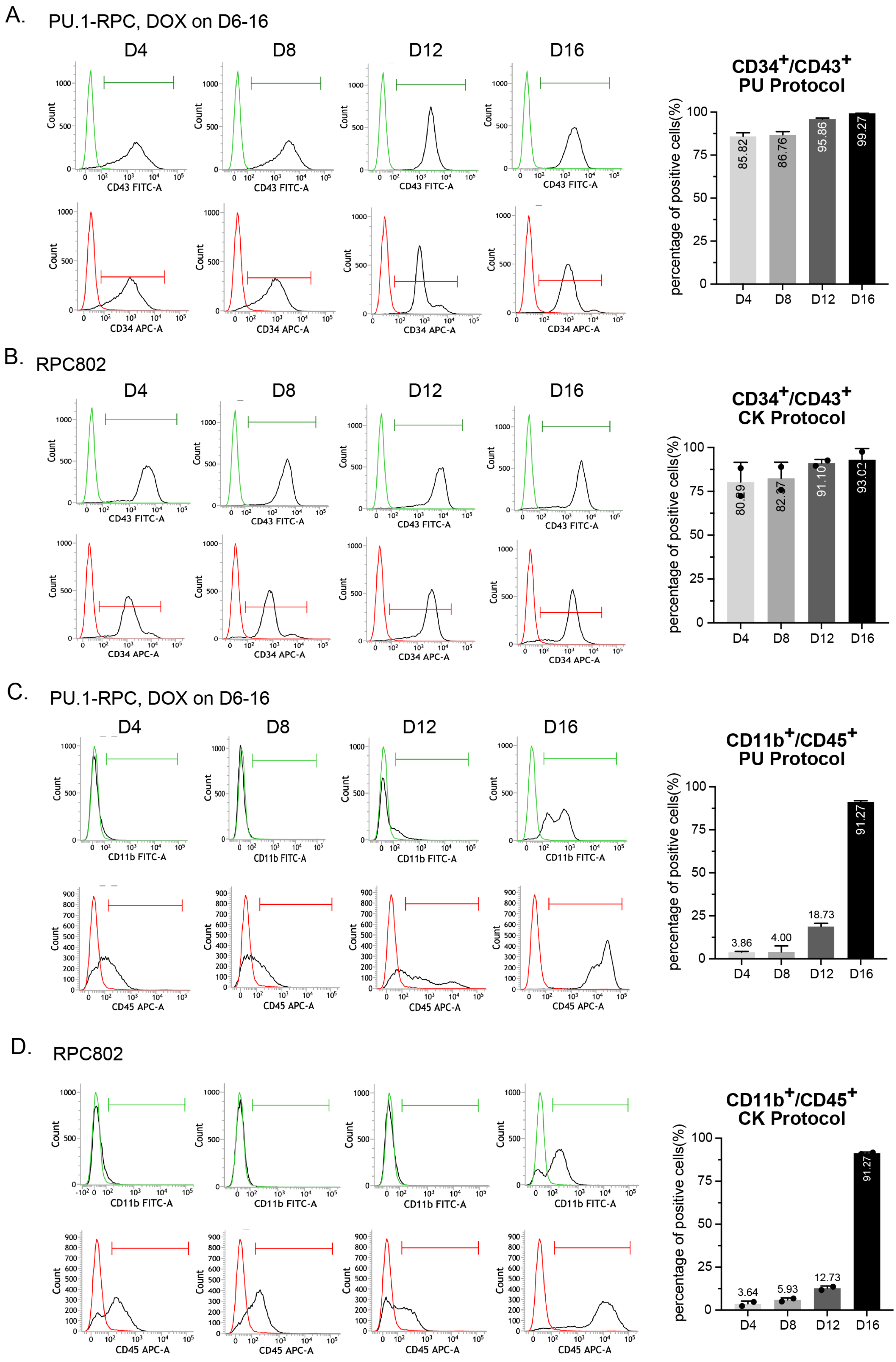
A.
PU.1-RPC, DOX on D6-16
D4
D8
D12
D16
Count
CD43 FITC-A
CD34 APC-A
CD34+/CD43+
PU Protocol
percentage of positive cells(%)
85.82
86.76
95.86
99.27
B.
RPC802
CD34+/CD43+
CK Protocol
80.89
82.97
91.10
93.02
C.
PU.1-RPC, DOX on D6-16
CD11b FITC-A
CD45 APC-A
CD11b+/CD45+
PU Protocol
3.86
4.00
18.73
91.27
D.
RPC802
CD11b+/CD45+
CK Protocol
3.64
5.93
12.73
91.27

Supplementary Fig. 6 PU-protocol has a stronger ability to induce cells to the primitive hematopoietic lineage. (Related to Fig.3 and Supplementary Fig.5) Ratios of positive cells of hematopoietic progenitor cell markers (A, B: CD43 and CD34) and myeloid cell markers (C, D: CD11b and CD45) were analyzed by flow cytometry. The ratio was not changed significantly between PU-protocol (A, C) and CK-protocol (B, D). Color curves in histogram indicated isotype control.

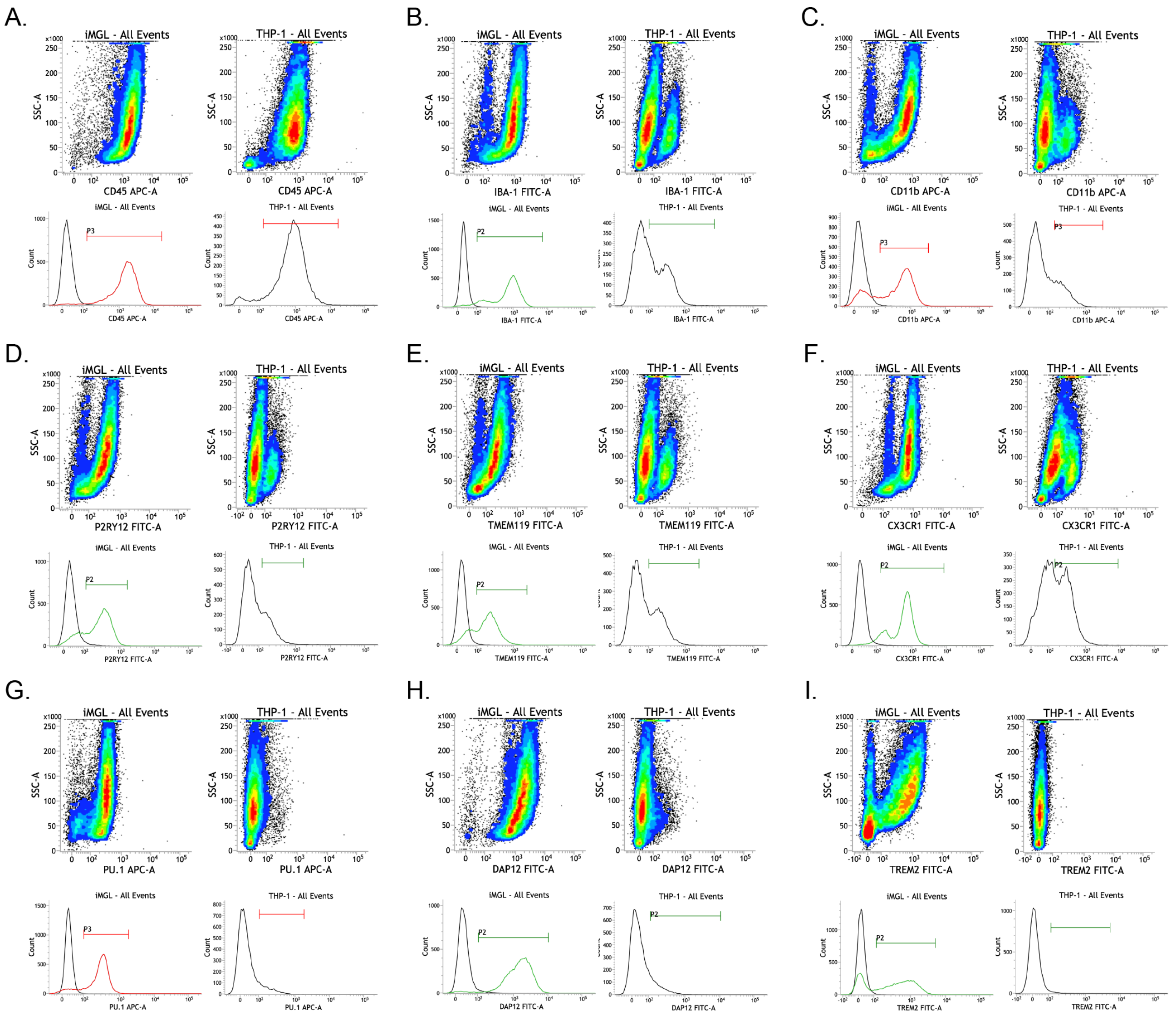

Supplementary Fig. 7 Purity of hiMGLs analyzed by flow cytometry. After re-plated on culture dishes for one week, hiMGLs were stained with microglia-specific markers or myeloid cell markers, then analyzed by flow cytometry. A human monocyte cell line, THP-1 cells, was used as a comparative cell population to confirm the potency of antibodies. Color curves in histogram indicated isotype control.

(A) The myeloid lineage marker CD45 was equally expressed in hiMGLs and THP-1.

(B-F) Microglia specific markers, IBA1 (B), CD11b (C), P2RY12 (D), TMEM119 (E), and CX3CR1 (F) were expressed by all hiMGLs cells, while only a small population of THP-1 cells expressed them.

(G) The major transcription factor of microglia, PU.1, was expressed by most hiMGLs cells, while almost none THP-1 cells expressed PU.1.

(H, I) The AD risk gene products, DAP12 and TREM2, were expressed by most hiMGLs, while few THP-1 cells were positive for both proteins.

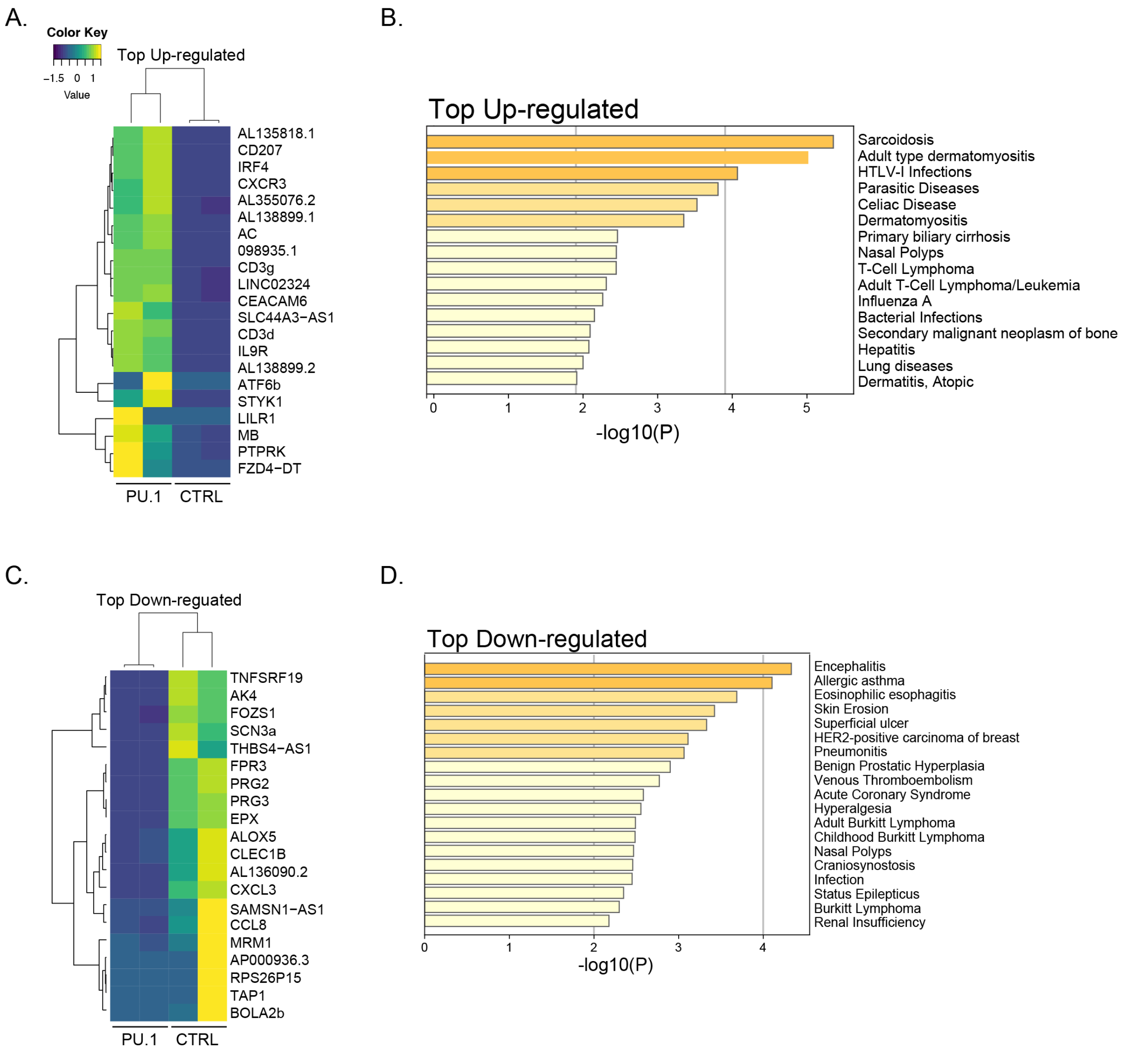

Supplementary Fig. 8 Gene expression level modestly varied between CK- and PU-protocol.

(A) Heatmap showing the top 20 genes that were upregulated by PU.1 overexpression.

(B) GO term enrichment analysis indicated that the genes upregulated by PU.1 overexpression were mostly related to pathogen response.

(C) Heatmap showing the top 20 genes that were downregulated by PU.1 overexpression.

(D) GO term enrichment analysis indicated that genes downregulated by PU.1 overexpression were mostly related to inflammation. Together with (B), the transcriptome profile showed that PU.1 overexpression would not alter the characteristics of microglia in the central nervous system.

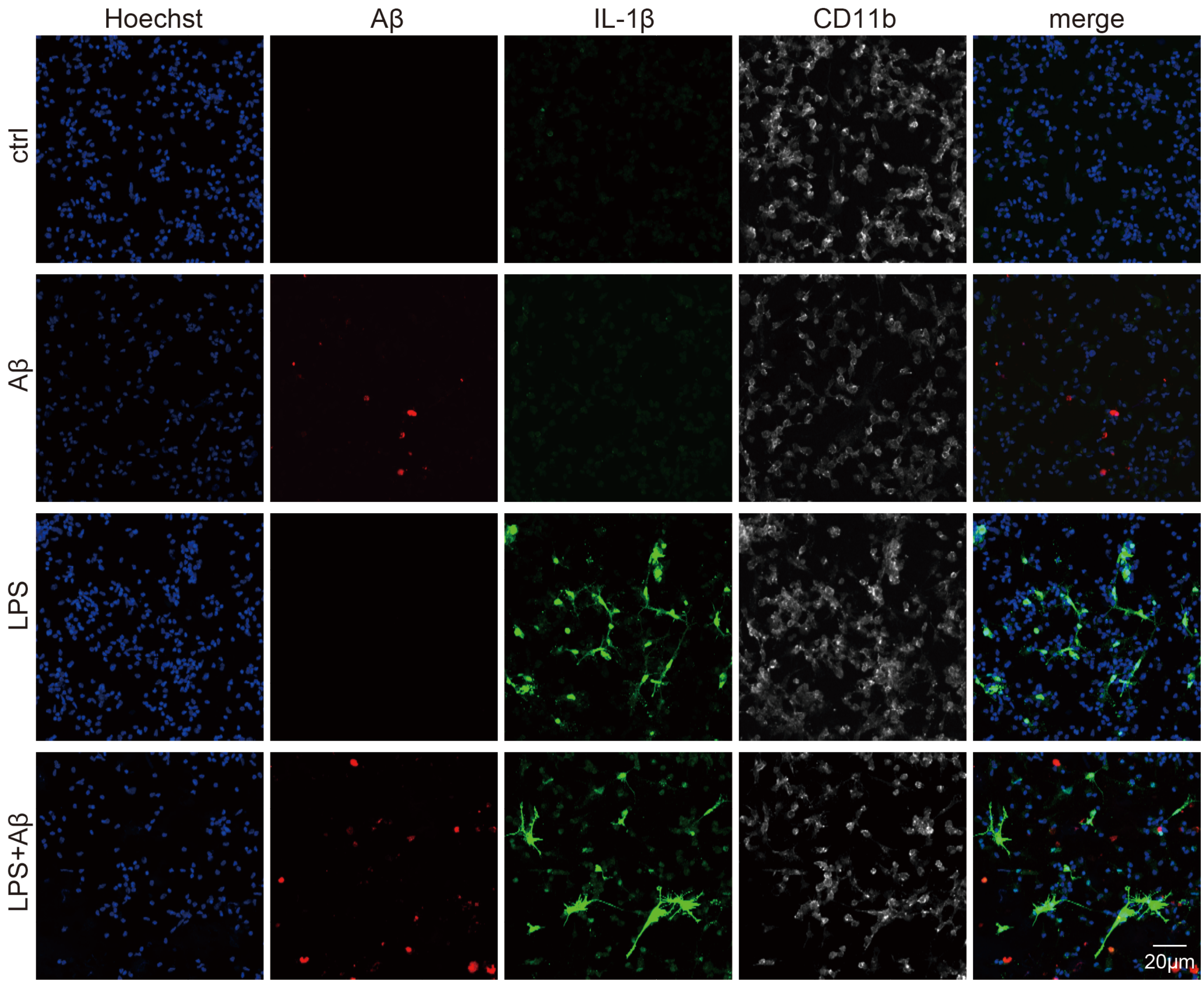

Supplementary Fig. 9 hiMGLs were able to form the inflammasome in response to LPS or Aβ peptide stimulation. Immunostaining images demonstrated that IL-1β expression was induced by LPS treatment, but not Aβ peptide. Scale bar = 20 μm.

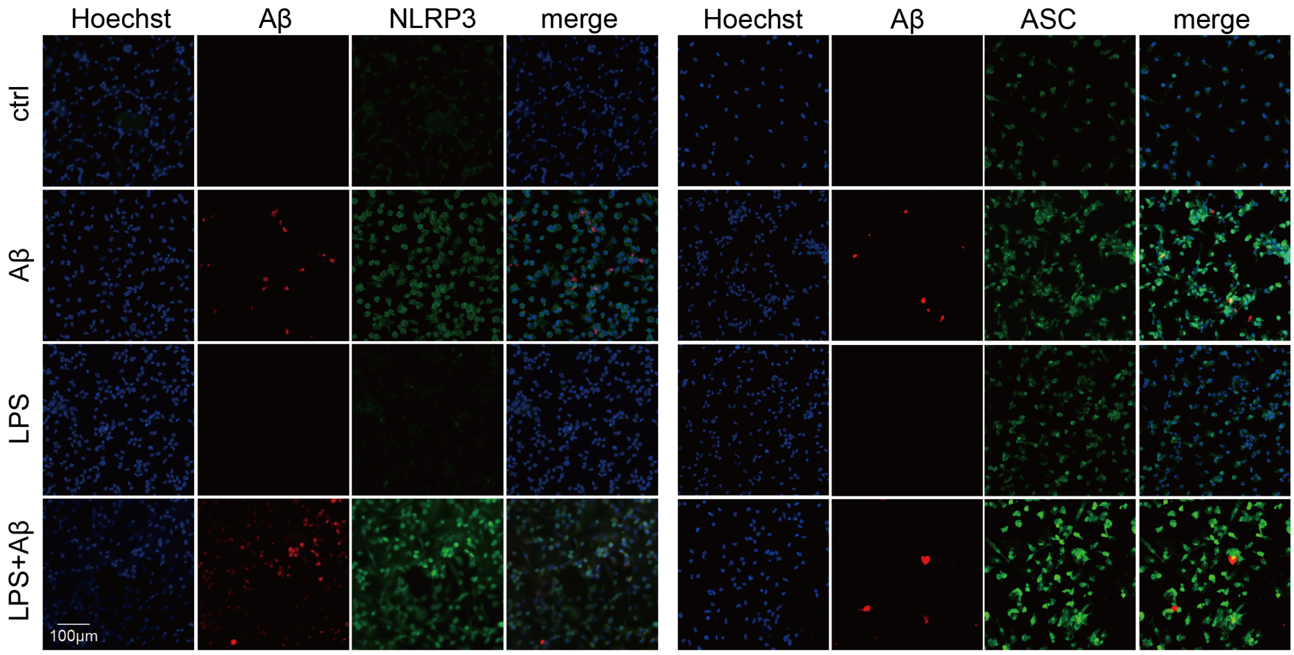

Supplementary Fig. 10 Aβ peptide, but not LPS alone, was able to stimulate the formation of inflammasome. (Related to Fig. 5D, supplementary Fig. 9) Although LPS alone was able to stimulate the expression of IL-1β, inflammasome formation was not stimulated by LPS alone. While LPS treatment followed by incubation together with Aβ peptide has induced the expression of NLRP3 and ASC, Aβ peptide alone was also able to upregulate the expression of NLRP3 and ASC, despite weaker expression without pre-treatment of LPS. Scale bar = 100 μm.

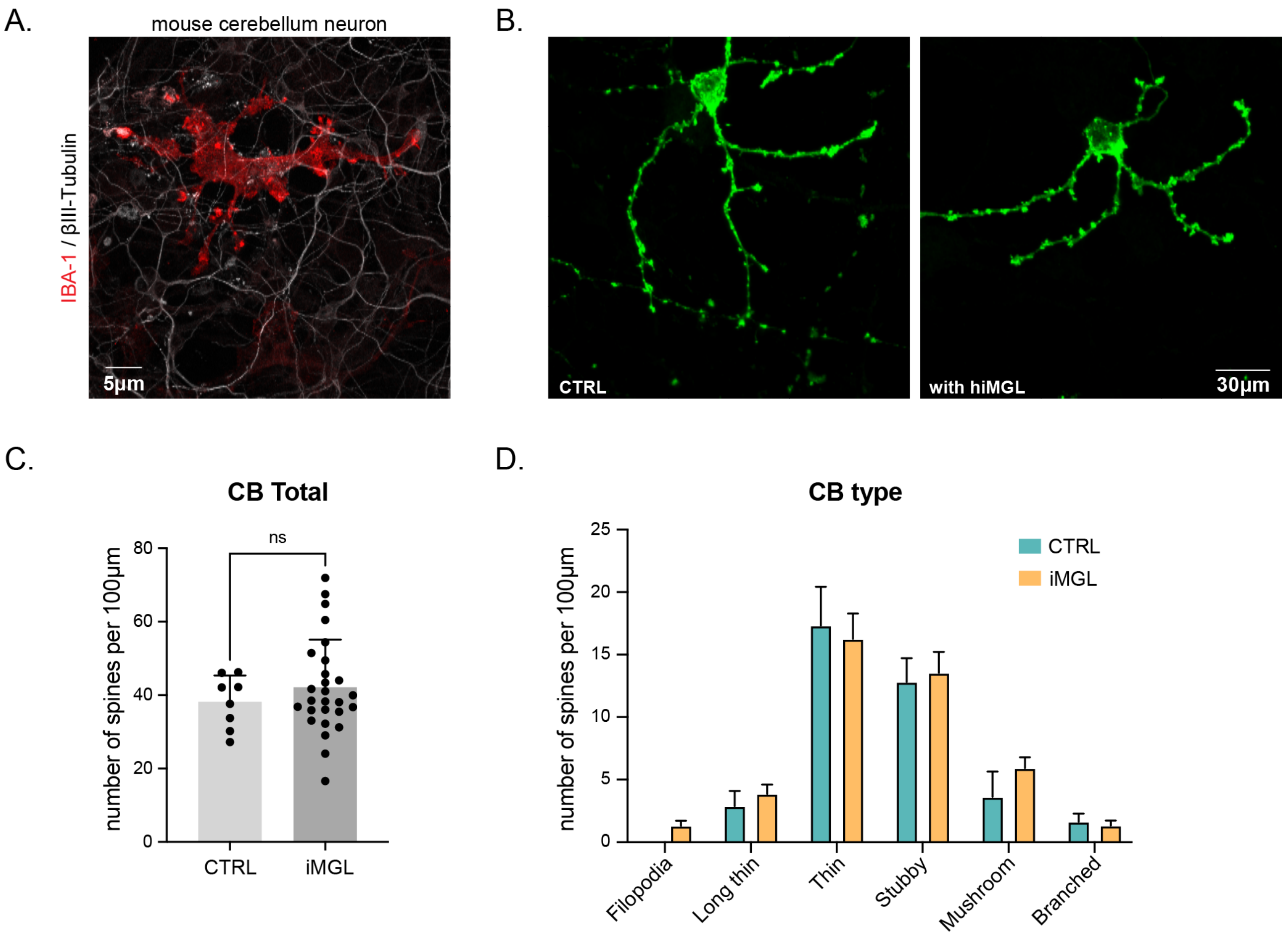

Supplementary Fig. 11 No significant differences in dendritic spines were detected between primary neurons from mouse cerebellum co-cultured with hiMGLs or in monoculture.

(A) hiMGLs showed a more ramified morphology after co-culture with cerebellum neurons. Scale bar = 5 μm.

(B) Primary granule neurons were transfected with β-actin -GFP plasmid. This morphological difference was not observed with or without hiMGL co-culture. Scale bar = 30 μm.

(C) There was no significant difference in the number of spines between the single culture and the co-culture system. (n = 8 in single culture, 27 in co-culture; 3 independent experiments). ns, not significant. All data are expressed as mean ± SEM.

(D) Spines were counted according to different morphological groups. No significant differences were detected.
